# TrkA of Streptococcus mitis CCUG31611 binds cyclic di-adenosine monophosphate and is required for growth in low potassium conditions

**DOI:** 10.1099/mic.0.001597

**Published:** 2025-08-14

**Authors:** Kristina Vevik, Biramitha Sribasgaran, Kun Cai, Ali-Oddin Naemi, Håkon Pedersen Kaspersen, Silvio Uhlig, Ole Andreas Økstad, Roger Simm

**Affiliations:** 1 Institute of Oral Biology, University of Oslo, 0316 Oslo, Norway; 2 Norwegian Veterinary Institute, 1433 Ås, Norway; 3 Nordic Institute of Dental Materials, 0855 Oslo, Norway; 4 Department of Pharmacy, University of Oslo, 0316 Oslo, Norway; 5 Department of Biosciences, University of Oslo, 0316 Oslo, Norway

**Keywords:** biofilm, cyclic di-adenosine monophosphate (c-di-AMP), DNA damage stress, potassium transport, *Streptococcus mitis*, TrkA family protein

## Abstract

Cyclic di-adenosine monophosphate (c-di-AMP) is a bacterial second messenger regulating many physiological processes in bacteria. In the oral commensal species *Streptococcus mitis*, c-di-AMP is involved in regulating metabolism, growth, colony morphology, chain length, biofilm formation and DNA stress tolerance. However, no c-di-AMP-regulated effector proteins have yet been characterized in *S. mitis*. In this study, we first show that a Δ*cdaA* mutant, unable to produce c-di-AMP, grows slowly under low environmental potassium conditions. Growth of the *cdaA* mutant was not restored by reintroducing *cdaA* in the original locus (KB*cdaA*). Whole-genome sequencing of multiple KB*cdaA* isolates revealed secondary mutations in a putative potassium transporter. The mutations were predicted to result in the truncation of the protein or the alteration of a conserved glycine residue essential for selective potassium uptake, disrupting protein function. A Δ*pde2* mutant overproducing c-di-AMP survived poorly under high environmental sodium concentrations. We then characterized the potassium transporter regulator protein TrkA. Biochemical analyses of the purified recombinant TrkA protein revealed that it specifically binds c-di-AMP with high affinity *in vitro*. Using deletion mutants of *trkA*, we demonstrate that TrkA is essential for growth under low environmental potassium conditions. Ultra-high-performance liquid chromatography coupled to tandem mass spectrometry revealed lower c-di-AMP concentration in the Δ*trkA* mutant compared to the WT. This was not due to transcriptional regulation of the expression of the c-di-AMP turnover proteins CdaA, Pde1 or Pde2. C-di-AMP production is not affected by the extracellular potassium concentrations under the conditions tested. We also demonstrate a potential role of TrkA in UV stress tolerance but do not characterize the mechanism in this study.

## Data Summary

The analysed whole-genome sequencing data have been deposited to European Nucleotide Archive (ENA) with project accession number PRJEB87292. The detailed list with accession numbers for each analysed bacterial isolate deposited to ENA can be found in Table S7, (available in the online version of this article).

## Introduction

Cyclic di-adenosine monophosphate (c-di-AMP) is a secondary signalling molecule found in bacteria and archaea [1,3]. Since its discovery by Witte *et al*. [4], many studies have shown that c-di-AMP is linked to complex cellular processes in bacteria. C-di-AMP is involved in controlling DNA repair [4], growth [5], biofilm formation [6], osmoregulation [7], metabolism [8,11], susceptibility to antibiotics [12] and virulence [13]. In addition, c-di-AMP secreted by bacteria can act in microbe–host interactions [14,15].

In our previous studies, we identified three putative c-di-AMP turnover proteins *in silico* in the oral commensal bacterium *Streptococcus mitis* CCUG31611 and established the enzymatic activity of these three proteins *in vitro* [16]. CdaA is so far the only identified diadenylate cyclase synthesizing c-di-AMP in *S. mitis*, and Pde1 and Pde2 are the only known phosphodiesterases that degrade c-di-AMP [16]. In line with this, the ∆*cdaA* mutant does not produce c-di-AMP, and the concentration of c-di-AMP is increased in the ∆*pde1* and ∆*pde2* mutants [16]. Furthermore, we determined the role of c-di-AMP in the physiology of *S. mitis* using knockout mutants of *cdaA*,* pde1* or *pde2* [16,17]. The ∆*cdaA* mutant grows in long chains, displays reduced biofilm formation and increased DNA stress tolerance. The ∆*pde1* mutant grows in shorter chains and displays reduced DNA stress tolerance. The ∆*pde2* mutant has reduced glucose metabolism, grows slowly and in short chains and displays small colony morphology on agar plates. The ∆*pde2* mutant also exhibits slightly reduced biofilm formation and reduced DNA stress tolerance [16,17].

C-di-AMP signalling is dependent on receptor molecules to convert information into cellular responses. Proteins and riboswitches have been identified as c-di-AMP receptors [18,19]. Riboswitches are RNA molecules that directly bind to small ligands to regulate gene expression [19,22]. C-di-AMP-specific riboswitches have so far not been identified in streptococci [19]. C-di-AMP binding to protein receptors can alter protein activity, either directly as an allosteric regulator of protein function or indirectly via altered protein–protein interactions [23,24].

Several c-di-AMP-specific protein receptors identified in different bacteria are involved in regulating potassium homeostasis and include potassium import proteins with associated gating proteins that directly control intracellular potassium concentrations [25,27], two-component systems controlling the expression of a potassium importer and a potassium exporter [28,29]. C-di-AMP has also been shown to regulate osmolyte transport by directly binding to the *Lactococcus lactis* glycine betaine transporter OpuA [30] or indirectly by binding to the transcriptional repressor BusR which in turn downregulates the transcription of a cytoplasmic protein BusA being part of an ABC osmolyte transporter BusAB in *Streptococcus agalactiae* [31]. The c-di-AMP-binding protein CbpB is involved in crosstalk with the (p)ppGpp pathway [23]. The carnitine transporter OpuCA, the pyruvate carboxylase (PycA) and the DNA recombinase RecA have also been shown to bind c-di-AMP and regulate carnitine export, oxaloacetate production and DNA repair, respectively [10,31, 32]. In addition, the master regulator DasR of *Saccharopolyspora erythraea* was shown to specifically bind c-di-AMP and negatively regulate the transcription of genes involved in the *N*-acetylglucosamine (GlcNAc) catabolism [33].

Four putative c-di-AMP-specific receptors (TrkA, KtrA, KimA and CbpB) have been identified in *S. mitis* CCUG31611 [34], but none of them have so far been characterized. In this study, we demonstrate that TrkA from *S. mitis* binds c-di-AMP and is essential for growth under low potassium concentrations. We also analyse the role of TrkA in other c-di-AMP-mediated phenotypes and demonstrate a potential role of TrkA in UV stress tolerance.

## Methods

### Identification and *in silico* characterization of a putative c-di-AMP-binding protein

The c-di-AMP-binding protein CabP from *Streptococcus pneumoniae* (SPD_0077; GenBank: ABJ54144.1) [25,35] was used as a query in a protein blast (blastp) search to identify putative c-di-AMP-binding proteins in *S. mitis* CCUG31611 (GenBank: CP028414.1). A multiple sequence alignment (MSA) of TrkA-like proteins and KtrA proteins (Table 1) was performed in Jalview v.2.11.2.6 with the Clustal Omega algorithm and default parameters [36]. Pairwise alignment of individual RCK domains was compared to *S. mitis* TrkA by EMBOSS Needle [37], and the sequence identity percentages are summarized in Table S1. Protein domain analysis was performed using InterProScan with default parameters [38]. The presence of a signal peptide and the prediction of protein subcellular localization were performed with default parameters in SignalP v.6.0 [39] and Gpos-mPLoc [40], respectively.

**Table 1.** Proteins used in the blastp searches and MSA

Strain name	GenBank ID	Locus name
***Streptococcus agalactiae* NEM316**	CAD47337.1	GBS1678
***Streptococcus gallolyticus* UCN34**	CBI14323.1	Gallo_1832
***Streptococcus mutans* UA159**	AAN59208.1	SMU_1562
***Staphylococcus aureus* subsp. *aureus* USA300_FPR3757**	ABD22474.1	SAUSA300_0988
***Streptococcus pneumoniae* D39**	ABJ54144.1	SPD_0077
**Strain name**	**Protein/protein domain**	**PDB ID**
***Bacillus subtilis* strain 168**	KtrA	KJ91
***Staphylococcus aureus* 08BA02176**	KtrA RCK_C domain	4XTT

### Bacterial strains and growth conditions

*S. mitis* CCUG31611, in this study referred to as WT, was used as a reference in all experiments for knockout mutants of diadenylate cyclase (∆*cdaA*), phosphodiesterase 1 (∆*pde1*), phosphodiesterase 2 (∆*pde2*), a gene encoding a putative protein involved in K^+^-transport (∆*trkA*), their double knockout mutants (∆*cdaA*∆*trkA*, ∆*pde1*∆*trkA* and ∆*pde2*∆*trkA*) and their respective knock-back (KB) strains (KB*cdaA*, KB*pde1*, KB*pde2*, KB*trkA*, ∆*cdaA*KB*trkA*, ∆*pde1*KB*trkA* and ∆*pde2*KB*trkA*). The strains used in this study are summarized in Table 2, and primers used for their construction can be found in Table S2. If not specified otherwise, *S. mitis* was grown in tryptone soy broth (TSB; Oxoid) statically at 37 °C and 5% CO_2_. For the experiments, all *S. mitis* strains used in this study were grown until mid-log phase (OD_600_≈0.5) and stored at −80 °C in 15% glycerol as pre-cultures. The *S. mitis* pre-cultures were inoculated at a 1 : 10 ratio in TSB and grown until mid-log phase (OD_600_≈0.5) for use in experiments. The *S. mitis* strains were also grown on blood agar plates [45 g l^−1^ Blood Agar Base No. 2 (ISO); VWR and 5 % v/v Sheep Blood (defibrinated); Thermo Fisher Scientific] incubated at 37 °C and 5% CO_2_. The *Escherichia coli* DH5*α* and BL21 (DE3) strains were used for cloning and protein expression, respectively. They were grown in lysogeny broth [LB; Bacto Tryptone (10 g l^−1^; Oxoid), Bacto Yeast Extract (5 g l^−1^; Becton, Dickinson and Company), NaCl (10 g l^−1^; Merck)] or on LB agar containing bacteriological agar (15 g l^−1^; VWR) at 37 °C. LB medium and agar were supplemented with 50 µg ml^−1^ kanamycin (Sigma-Aldrich) for selection of *E. coli* carrying the pET30b and pET30b_TrkA-N-His plasmids (Table 2).

**Table 2.** Bacterial strains, mutants and plasmids used in this study

Strain name	Description	Origin	Reference
***S. mitis* CCUG31611**	Type strain *S. mitis*, corresponding to NCTC 12261 and ATCC 49456		CCUG
***S. mitis* Δ*cdaA***	Markerless in-frame deletion of SM12261_1350	*S. mitis* CCUG31611	[16]
***S. mitis* Δ*pde1***	Markerless in-frame deletion of SM12261_1779	*S. mitis* CCUG31611	[16]
***S. mitis* Δ*pde2***	Markerless in-frame deletion of SM12261_1122	*S. mitis* CCUG31611	[16]
***S. mitis* Δ*trkA***	Markerless in-frame deletion of SM12261_0089	*S. mitis* CCUG31611	This study
***S. mitis* Δ*cdaA*Δ*trkA***	Markerless in-frame deletion of SM12261_1350 and SM12261_0089	*S. mitis* Δ*cdaA*	This study
***S. mitis* Δ*pde1*Δ*trkA***	Markerless in-frame deletion of SM12261_1779 and SM12261_0089	*S. mitis* Δ*pde1*	This study
***S. mitis* Δ*pde2*Δ*trkA***	Markerless in-frame deletion of SM12261_1122 and SM12261_0089	*S. mitis* Δ*pde2*	This study
***S. mitis* KB*cdaA***	SM12261_1350 re-introduced into the original locus	*S. mitis* Δ*cdaA*	[16]
***S. mitis* KB*pde1***	SM12261_1779 re-introduced into the original locus	*S. mitis* Δ*pde1*	[16]
***S. mitis* KB*pde2***	SM12261_1122 re-introduced into the original locus	*S. mitis* Δ*pde2*	[16]
***S. mitis* KB*trkA***	SM12261_0089 re-introduced into the original locus	*S. mitis* Δ*trkA*	This study
***S. mitis* Δ*cdaA*KB*trkA***	Markerless in-frame deletion of SM12261_1350 and SM12261_0089 re-introduced into the original locus	*S. mitis* Δ*cdaA* Δ*trkA*	This study
***S. mitis* Δ*pde1*KB*trkA***	Markerless in-frame deletion of SM12261_1779 and SM12261_0089 re-introduced into the original locus	*S. mitis* Δ*pde1* Δ*trkA*	This study
***S. mitis* Δ*pde2*KB*trkA***	Markerless in-frame deletion of SM12261_1122 and SM12261_0089 re-introduced into the original locus	*S. mitis* Δ*pde2* Δ*trkA*	This study
***E. coli* DH5*α***	Strain used for cloning and as a negative control for intracellular nucleotide concentration		Thermo Fisher Scientific
***E. coli* TOP10**	Strain used for cloning and as a negative control for intracellular nucleotide concentration		Thermo Fisher Scientific
***E. coli* BL21 (DE3**)	Strain used for the expression of recombinant proteins		Thermo Fisher Scientific
**Plasmid name**	**Description**	**Origin**	**Reference**
**pET30b**	Expression vector with IPTG-inducible T7 promoter; Kan^R^		Novagen
**pET30b_TrkA-N-His**	pET30b-vector with the *trkA* gene ORF (SM12261_RS00450; 1–221 aa) containing an N-terminal 6× His-tag; Kan^R^	pET30b	This study

### Cloning and expression of *trkA*

The entire ORF of *trkA* (SM12261_0089) was amplified from the genome of *S. mitis* CCUG31611 using primers RS450 and RS435 (Table S2). The amplicon was cloned into the pET30b vector between restriction sites *NdeI* and *HindIII* cleaved with FastDigest^™^ enzymes (Thermo Fisher Scientific) and ligated with the Rapid DNA Ligation Kit (Thermo Fisher Scientific). The recombinant plasmid DNA was amplified in *E. coli* DH5*α* and later sequenced at Eurofins Genomics (Konstanz, Germany). The list of primers used for cloning and sequencing is available in Table S2. The pET30b_TrkA-N-His construct was transformed into *E. coli* BL21 (DE3) for protein expression. An overnight culture was inoculated at a 1 : 100 ratio into LB supplemented with kanamycin (50 µg ml^−1^) and incubated at 37 °C, 220 r.p.m. until the culture reached the mid-log phase (OD_600_=0.4–0.5). The expression of recombinant TrkA was induced with 1 mM IPTG (Merck). After induction, the bacterial culture was incubated at 37 °C for 3 h at 220 r.p.m.

### Purification of recombinant TrkA protein

Bacterial cells were collected at 4,000 ***g*** for 10 min at 4 °C and later resuspended in equilibration buffer (150 mM KCl, 20 mM HEPES, 10 mM imidazole, pH 7.5) containing 1× EDTA-free SIGMAFAST^™^ Protease Inhibitor Cocktail (Merck), 0.1 mg ml^−1^ lysozyme (Sigma-Aldrich), 10 µg ml^−1^ DNaseI (Merck), 10 mM MgCl_2_ (Sigma-Aldrich) and 10 mM MnCl_2_ (ThermoFisher). The mixture was incubated for 30 min on ice and later lysed using a sonicator. The resulting whole cell lysate was centrifuged at 12,000 ***g*** for 10 min at 4 °C, and the supernatant was filter-sterilized with a 20 µm syringe filter. The filtered supernatant was used in the purification process.

The first purification step was done with a Pierce™ Disposable 10 ml Polypropylene Column (Thermo Fisher Scientific) and reusable HisPur^™^ Ni-NTA resin (Thermo Fisher Scientific) according to the manufacturer’s instructions. Buffers with an increasing imidazole concentration (10, 25, 50 and 150 mM) were used for column wash. The TrkA protein was eluted with elution buffer (150 mM KCl, 20 mM HEPES, 250 mM imidazole; all Sigma-Aldrich, pH 7.5). The fractions containing TrkA were collected and concentrated in an Amicon® Ultra Centrifugal Filter Unit (Millipore) with a molecular weight (MW) cut-off of 3 kDa at 5,000 ***g*** and 4 °C for 2 h.

The second purification step involved size exclusion chromatography using a Sepharose 6 Increase 10-300 GL column (GE Healthcare) and ÄKTA pure™ system (Cytiva Life Sciences). The column was equilibrated, and TrkA protein was eluted with running buffer (150 mM KCl, 20 mM HEPES, pH 7.5) over 1.5 column volumes at a flow rate of 0.2 ml min^−1^ according to the manufacturer’s instructions. The isolated protein was stored at −80 °C in the same running buffer with the addition of 10% glycerol (storage buffer: 150 mM KCl, 20 mM HEPES, 10% glycerol, pH 7.5). The purity of the protein was determined by running the sample on a 4–20% acrylamide SDS-PAGE gel followed by Coomassie staining. The protein identity was confirmed by detection of 6×His tag on Western blot (WB) with a primary mouse anti-His-tag monoclonal antibody (Thermo Fisher Scientific) and secondary donkey anti-mouse HRP-conjugated antibody (Thermo Fisher Scientific). The WB signal was detected with Pierce^™^ ECL Western Blotting Substrate (Thermo Fisher Scientific), and the final concentration of the purified protein was measured with DC Protein Assay (Bio-Rad).

### Determination of quaternary protein structure

The purified TrkA protein was run in the same chromatography column, system and buffer as explained for the second purification step. In addition, a protein standard (Gel Filtration Markers Kit for Protein Molecular Weights 12,000–200,000 Da; Sigma-Aldrich) was run to determine the protein size. To estimate the MW of *S. mitis* TrkA recombinant protein in solution, a standard curve was used where the logarithm of the MW of reference proteins was plotted against the ratio between the elution volume (V_E_) and void volume (V_0_) (logMW vs V_E_/V_0_).

### Ligand binding assays – nano differential scanning fluorimetry

Serial twofold dilutions of chosen nucleotideligands [c-di-AMP (Biolog), pApA (Biolog), ATP (Sigma-Aldrich), AMP (Sigma-Aldrich) and c-di-GMP (Biolog)] were prepared in 16 PCR tubes in MilliQ H_2_O. The concentration of ligands in the first tube was 500 µM. Each ligand dilution was added to TrkA prepared in storage buffer (150 mM KCl, 20 mM HEPES, 10% glycerol, pH 7.5) to a final concentration of 500 nM in all 16 tubes. The total reaction volume in each tube was 20 µl. Reactions were incubated at room temperature (RT) for 5 min and then loaded into the glass capillaries (Prometheus NT.48 Capillaries nano DSF) and loaded onto the Prometheus NT.48 nanoDSF machine (NanoTemper). Additional samples of TrkA solution or ligand solutions alone were used as controls for the inherent fluorescence signal. The experiment was repeated twice, each with two technical replicates. The results were analysed with the FoldAffinity online software to estimate the ‘apparent’ K_d_-values [41,42]. In the analysis, the approach of Bai *et al*. [43] and Short *et al*. [44] was used. The K_d_-values with the lowest error at a certain isothermal fitting temperature were chosen.

### Generation of Δ*trkA* knockout mutants through markerless gene deletion

For the construction of knockout mutants, we adapted the method developed by Salvadori *et al*. [45]. The strain to be mutated was grown from a pre-culture diluted 1 : 10 in the C+Y_YB_ medium for 2 h at 37 °C in a 5% CO_2_ atmosphere [46]. The *S. mitis* competence-stimulating peptide (300 nM) and 200 ng of the purified DNA amplicon were added to the bacterial culture (300 µl). The reaction mixture was incubated at 37 °C for 3 h on a heating block. After incubation, serial dilutions (10^0^–10^−7^) were prepared in 1× PBS [10× PBS: 1 M Na_2_HPO_4_ (Millipore), 0.018 M KH_2_PO_4_ (Sigma), 1.54 M NaCl (Sigma-Aldrich) and 0.027 M KCl (Sigma-Aldrich)], and 25 µl of each dilution was plated on blood agar for later selection of single colonies. The plates were incubated for 48 h at 37 °C and 5% CO_2_. Single colonies were picked and inoculated on a new blood agar plate and used for colony PCR to detect colonies with the intended mutations. The primers used for the construction and screening of colonies are listed in Table S2. An erythromycin resistance cassette was used as a control for the transformation efficiency as described by Salvadori *et al*. [45].

### Whole-genome sequencing of *S. mitis* strains and bioinformatic analyses

*S. mitis* overnight cultures were put on ice for 10 min and collected at 5,000 ***g*** for 10 min at 4 °C. The genomic DNA was isolated with MasterPure™ Gram-Positive DNA Purification Kit (Epicentre™) according to the manufacturer’s instructions. The final DNA concentrations were measured with NanoDrop™ 2000c (Thermo Scientific™) and Qubit™ 4 Fluorometer (Invitrogen). To assess sample quality, each DNA sample (2 µl) was run on a 1% agarose gel at 75 V for 30 min. DNA samples were stored at −80 °C before sending to Eurofins Genomics for whole-genome sequencing with Illumina NovaSeq X+ (150 bp reads, ~5 million read pairs). Quality control and assembly of the sequenced genomes were performed by the Assemblage pipeline [47]. Briefly, initial quality control was done by running FastQC v.0.12.1 [48] and MultiQC v.1.14 [49], before trimming the reads using Trim-galore v.0.6.10 [50], using a phred-score cutoff of 15, error rate of 0.1 and minimum read length of 20 bp. The reads were checked for contamination by using Kraken2 v.2.1.3 [51] and the pre-built Minikraken V2 database (updated April 2019). Trimmed reads were assembled with Unicycler v.0.5.0 [52], using a minimum contig length of 500 and a depth filter of 0.25. All assemblies were quality controlled with Quast v.5.2.0 [53]. The WT genome was annotated with Bakta v.1.9.2 [54] using a reference genome (accession number GCF_000148585.2_ASM14858v3) as a reference for the annotation. Snippy v.4.6.0 [55] was used to identify differences between the annotated WT genome and the rest of the samples (Table S7), using the trimmed reads from the assembly step as input. blast v.2.12.0 [56] was used to search for the presence of *cdaA*, *pde1*, *pde2* and *trkA*. The sequences of these genes were extracted from the WT genome and used as a query in the blast search. In the blast search, the BLOSUM 62 matrix was used with *E*-value threshold set to 0.05.

### Nucleotide extraction from bacterial pellets

*S. mitis* pre-cultures were inoculated at a 1 : 10 ratio in TSB and grown to mid-log phase (OD_600_≈0.5) under standard conditions. The mid-log phase WT strain grown in TSB to be later tested for growth in chemically defined medium (CDM) (CDM with 1 or 10 mM KCl) was washed twice in CDM without KCl and centrifuged at 5,000 ***g*** for 10 min at RT. The cells were resuspended in CDM without KCl and inoculated into CDM media at a 1 : 10 ratio. They were grown to mid-log phase under standard conditions. After reaching the desired OD_600_-value, bacterial suspensions were centrifuged as described earlier in this paragraph. The supernatant was discarded, and pellets were stored at −20 °C until use. Pellets were thawed at RT and resuspended in 300 µl extraction buffer (v/v 40% acetonitrile, 40% methanol, 20% water; ULC/MS-CC/SFC grade; Biosolve). The pellets were incubated on ice for 15 min followed by heating at 95 °C for 10 min and cooling down again on ice for another 15 min. Samples were centrifuged for 15 min at 4,000 ***g*** at 4 °C, and supernatants were transferred into new Eppendorf tubes. The extraction procedure was repeated twice with 200 µl extraction buffer, and supernatants from the same sample were pooled into one tube (resulting in ~700 µl supernatant). Samples were stored at −20 °C overnight and centrifuged the next day (4,000 ***g***), and supernatants were transferred into new Eppendorf tubes [57,58]. The supernatants with extracted nucleotides were stored at −20 °C until use. Two *E. coli* strains (DH5*α* and TOP10) were used as negative controls. All cultures were prepared in triplicate.

### Quantification of c-di-AMP using ultra-high-performance liquid chromatography coupled to tandem mass spectrometry

Cyclic-di-AMP was determined and quantified using an isotope-dilution ultra-high-performance liquid chromatography coupled to tandem mass spectrometry (UHPLC-MS/MS) assay, following partial purification of extracts by solid-phase extraction. The validation of the analytical method, including the determination of other cyclic dinucleotides, is reported in a separate communication [59]. Cyclic-di-AMP was quantified in the supernatants that were prepared as described in the ‘Nucleotide extraction from bacterial pellets’ section. Briefly, 200 µl of each supernatant was diluted with 600 µl of 2% acetic acid (ULC/MS-CC/SFC grade; Biosolve) and applied to Oasis® WAX cartridges (30 mg, 1 ml; Waters, Milford, MA, USA) that had been equilibrated with 1 ml of methanol (ULC/MS-CC/SFC grade; Biosolve) followed by 2 ml of 2% acetic acid [58]. The columns were washed with 1 ml 2% acetic acid, followed by methanol/water (4 : 1, v/v). The columns were dried under vacuum, and samples were eluted with a solution of methanol/25% ammonia (4 : 1, v/v). The eluates were dried at 40 °C under a constant flow of nitrogen gas. The dried residues were resuspended in 200 µl of water (ULC/MS-CC/SFC grade; Biosolve) and transferred to chromatography vials with inserts, and 10 µl of an 840 nM ^15^N_10_-labelled c-di-AMP (Biolog) solution was added. Samples were stored at −20 °C until UHPLC-MS/MS analysis.

The bacterial extracts were analysed using a 6470A triple quadrupole mass spectrometer that was connected to a 1290 Infinity II UHPLC via a Jet Stream electrospray interface (all Agilent Technologies, Santa Clara, CA, USA). An Avantor^®^ ACE^®^ Excel C18-Amide column (100×2.1 mm i.d., 1.7 µm particles; Avantor, Inc., Radnor, PA, USA) was used for UHPLC, which was eluted using a mobile phase consisting of 5 mM ammonium acetate (A) and acetonitrile (B) at a flow rate of 0.35 ml min^−1^. The column was eluted isocratically for 2 min with 1% B, followed by linear gradient elution to 12% B over 6 min. The retention time of c-di-AMP under these conditions was 4.4 min. The lower limit of quantification of c-di-AMP was 3 nM.

Individual concentrations of c-di-AMP were normalized to total protein. Thus, the total protein content in the nucleotide-extracted bacterial pellets was determined using a modified Bradford assay [60]. In short, the bacterial pellets were resuspended in 300 µl of SDS sample buffer (250 mM Tris pH 8.0, 1% (w/v) SDS and 50 mM *β*-mercaptoethanol) and heated at 95 °C for 10 min. Aliquots (5 µl) of each sample were pipetted in triplicate into a transparent 96-well plate with a flat bottom (Thermo Fisher Scientific). Cydex Blue solution [250 µl; Bradford Protein Assay Reagent (Thermo Fisher Scientific) containing 2.5 mg ml^−1^ α-cyclodextrin] was added to the wells. The plates were shaken for 30 s and statically incubated for 10 min at RT. All assays included two sets of BSA standards that were prepared from a commercial concentrate (2.0 mg ml^−1^ in 0.9% NaCl with NaN_3_), which is part of the Pierce™ Bradford Protein Assay Kit (Thermo Fisher Scientific), with working range of 100–1,500 and 1–25 µg ml^−1^ prepared according to the manufacturer’s instructions.

###  Isolation of RNA and real-time quantitative PCR

To determine the transcriptional profiles of *trkA*, *cdaA*, *pde1* and *pde2* in various strains, bacterial cultures were grown and pellets were prepared as described in the ‘Nucleotide extraction from bacterial pellets’ section. For mRNA isolation from bacterial cells, the High Pure RNA-Isolation Kit (Roche) was used following the manufacturer’s instructions for bacterial RNA isolation. The RNA was quantified, and the quality was assessed with NanoDrop™ 2000c (Thermo Scientific™). cDNA was generated from total RNA using the First Strain cDNA Synthesis Kit (Thermo Fisher Scientific) as described by the manufacturer. Quantitative real-time PCR was performed using the PowerUP™ SYBR™ Green Master Mix (Thermo Scientific). DNA gyrase A was used as a reference gene. Relative expression levels of the target gene transcripts were then calculated by normalizing the levels of the specific RNA of each target gene with WT. All samples were compared, and relative fold changes in the samples were calculated using the 2^–ΔΔCt^ method.

###  Growth assay in TSB

*S. mitis* pre-cultures were inoculated at a 1 : 10 ratio in TSB and grown to mid-log phase (OD_600_≈0.5) under standard conditions. Bacterial suspensions were adjusted to an OD_600_-value of 0.001 with RT TSB. One hundred microlitres of aliquots were pipetted in duplicate into a 96-well plate with flat bottom wells together with TSB as a blank. Ninety-six-well plates were incubated at 37 °C in a plate reader (Cytation 3 Imaging Reader; Biotek) for 20 h (aerobic conditions). The OD_600_ measurements were done every 15 min, preceded by 15-s linear shaking. The cultures were tested in duplicate with three technical replicates pipetted from each culture.

### Growth assay in the modified CDM

For the growth of *S. mitis* in medium with defined potassium concentrations, we modified a recipe of a CDM (Tables S3 and S4) [61,62]. Four CDM media were prepared with different final concentrations and sources of potassium. One complete CDM medium (normal CDM) was prepared with potassium phosphates as a source of potassium (K_2_HPO_4_ and KH_2_PO_4_). The modified versions of the medium were first prepared potassium-free and were later added KCl to the final concentrations of 0, 1.11 and 11.11 mM. The final KCl concentrations in the assay were 0, 1 and 10 mM KCl in the modified media.

*S. mitis* pre-cultures were inoculated at a 1 : 10 ratio in TSB and grown to mid-log phase (OD_600_≈0.5) under standard conditions. The bacterial cells were centrifuged at 5,000 ***g*** for 10 min at RT and washed twice with RT CDM medium without potassium (CDM 0 mM KCl). The OD_600_ of bacterial cultures was adjusted to 0.1 with the same CDM medium. Each type of CDM medium, 100 µl for blanks and 90 µl for samples, was distributed into a flat and translucent 96-well microtitre plate. Ten microlitres of each bacterial suspension were pipetted into the wells with 90 µl of media in duplicate. The microtitre plate was covered with MicroAmp™ Optical Adhesive Film (Applied Biosystems, USA). The growth assay was performed at 37 °C under aerobic conditions, and OD_600_-values were registered over a 20-h period every 15 min preceded by 15-s linear shaking in a Cytation 3 Imaging Reader (Biotek). The experiments were repeated twice.

### Biofilm formation in 24-well plate

*S. mitis* pre-cultures were inoculated at a 1 : 10 ratio in TSB and grown to mid-log phase (OD_600_≈0.5). The OD-value of cultures was adjusted to 0.1 with TSB. Nine hundred microlitres of TSB and 100 µl of the desired bacterial suspension were pipetted into a translucent 24-well plate (final OD_600_=0.01) and incubated at 37 °C in 5% CO_2_ for 20 h. TSB (1 ml) was included in each plate as a blank, as well as 900 µl TSB with 100 µl WT strain serving as a reference strain. The assay was performed using different types of TSB where the pH was buffered with PBS and adjusted using HCl (non-adjusted pH, pH 5.5 and pH 7.2). After 20 h of incubation, the medium was carefully removed, and 1 ml of 0.1% Safranin solution (Sigma-Aldrich) was added to the wells and incubated at RT for 30 min. Safranin was removed, and biofilms were washed twice with PBS. Thirty per cent acetic acid (1 ml; VWR) was added to each well and incubated for 30 min at RT. Absorbance was measured at 530 nm in a plate reader (Cytation 3 Imaging Reader, Biotek). The experiment was repeated at least three times with three technical replicates for each strain, and the data were normalized against the WT strain of each experiment.

### MIC assay

Bacterial suspensions were adjusted to an OD_600_-value of 0.01, and 10 µl aliquots were pipetted into a 96-well plate with round-bottom wells. Ninety microlitres of either ciprofloxacin (Merck; 8.0–0.25 µg ml^−1^) or ampicillin (Merck; 0.0625–0.0039 µg ml^−1^) prepared as twofold serial dilutions in TSB were added to the wells. TSB and bacterial suspensions without antibiotics were used as assay controls. The 96-well plates were incubated for 24 h at 37 °C in a humidified atmosphere with 5% CO_2_. The MIC was determined as the lowest concentration of antibiotics inhibiting visual bacterial growth. The experiment was repeated four times.

### Osmotic stress assay

Pre-cultures were inoculated at a 1 : 5 or 1 : 10 ratio in TSB, and the OD_600_ of the mid-log phase cultures was adjusted to 0.3. The cultures were 10-fold serially diluted in PBS (10^−1^–10^−9^). The dilutions of each strain were spotted in duplicate (5 µl) onto the tryptic soy agar plates containing either 0 or 0.2 M NaCl and incubated at 37 °C with 5% CO_2_. C.f.u. values were counted after 48 h. The experiment was repeated at least three times, and the data were normalized against the WT strain. The data are presented as the relative ratio between the number of c.f.u. values on plates with and without NaCl.

### UV radiation assay

Mid-log phase cultures of *S. mitis* strains were adjusted to an OD_600_ of 0.3, and serial dilutions were made in TSB (10^0^–10^−6^). Aliquots (5 µl) of each dilution were spotted onto two separate blood agar plates in duplicate. One of the plates was used as a control, and the other was exposed to UV radiation of 2.5 mJ cm^2^. Both treated and untreated blood agar plates were incubated at 37 °C, 5% CO_2_ for 48 h. After incubation, c.f.u. values were counted, and the ratio between bacteria surviving UV radiation and total bacteria used in the experiment was calculated. All strains were normalized against the results with the WT strain.

### Statistical analysis

The statistical analyses and preparation of graphs were performed using GraphPad Prism 10.2.0 (392).

## Results

### Identification of putative c-di-AMP-binding protein TrkA in *S. mitis* CCUG31611

**Fig. 1.**
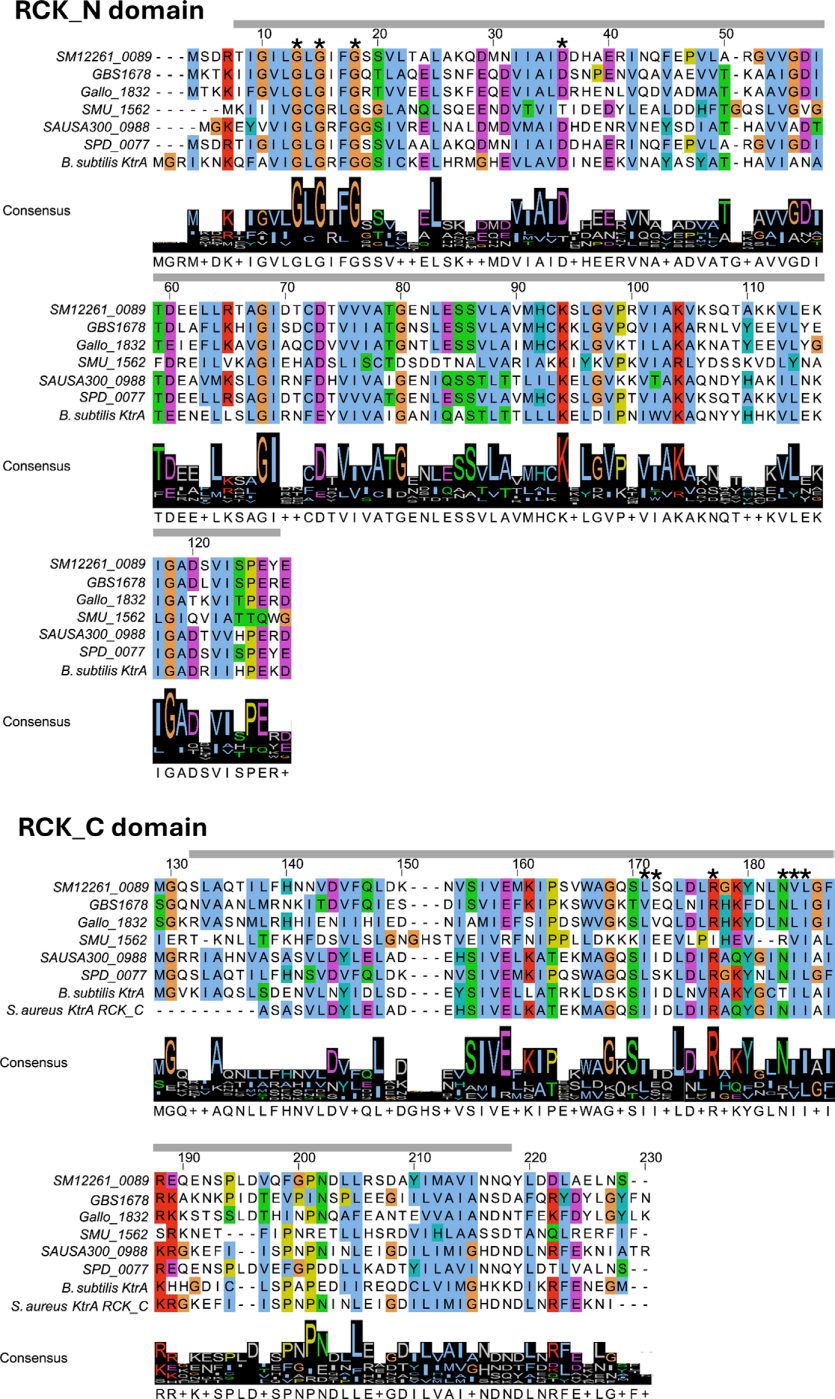
MSA of TrkA proteins. Amino acid (aa) sequences of individual RCK domains of the indicated proteins were aligned with the corresponding domains of TrkA from *S. mitis* CCUG31611 (SM12261_0089), *S. agalactiae* NEM316 (GBS1678), *Streptococcus gallolyticus* UCN34 (Gallo_1832), *Streptococcus mutans* UA159 (SMU_1562), *S. aureus* subsp. *aureus* USA300_FPR3757 (SAUSA300_0988), *S. pneumoniae* D39 (SPD_0077), *Bacillus subtilis* strain 168 (KtrA) and *S. aureus* 08BA02176 (KtrA RCK_C). Conserved aa residues are coloured, and the predicted RCK domains and functional motifs of TrkA are indicated above the alignment. Both the N-terminal (aa 5–132) and C-terminal (aa 140–221) RCK domains are indicated with grey lines. The aa present in the functional motifs with potential function in nucleotide binding are marked with asterisks.

blast searches conducted using the amino acid (aa) sequences of Trk-like proteins (Table 1), previously shown to bind c-di-AMP, against the *S. mitis* CCUG31611 genome (GenBank: CP028414.1) identified the two putative RCK_N (regulators of K^+^ conductance; also TrkA_N) domain proteins SM12261_0089 (TrkA; GenBank: QBZ10975.1) and SM12261_1578 (CabPB; GenBank: QBZ12372.1). TrkA displayed 91.86% identity and 100% coverage compared to SPD_0077, while SM12261_1578 displayed 97.1% identity and 100% coverage compared to SPD_0430 of *S. pneumoniae*. The gene encoding TrkA is located downstream of *trkH*, a gene encoding a putative cation transport family protein (GenBank: QBZ10974.1; gene SM12261_0088). Similarly, CabPB is located upstream of a gene encoding a putative cation transport family protein (GenBank: QBZ12373.1; gene SM12261_1579). In *S. pneumoniae*, the deletion of SPD_0077, but not SPD_0430, had an effect on growth under low potassium concentrations and under conditions stimulating genetic competence [25,63], and we selected the SPD_0077 homologue TrkA for further characterization in this study. The full-length TrkA contains 221 aa and is predicted to be a soluble cytosolic protein that consists of one RCK_N and one RCK_C domain (Figs1  2a). The MSA revealed that the RCK_N domain of *S. mitis* TrkA contains the characteristic [GXGXXGX_n_D/E] nucleotide-binding motif [64,67]. On the other hand, the aa demonstrated to form a nucleotide-binding motif in the RCK_C domain of KtrA from *Staphylococcus aureus* is less conserved among the proteins in the MSA (Fig. 1) [68,69].

**Fig. 2.**
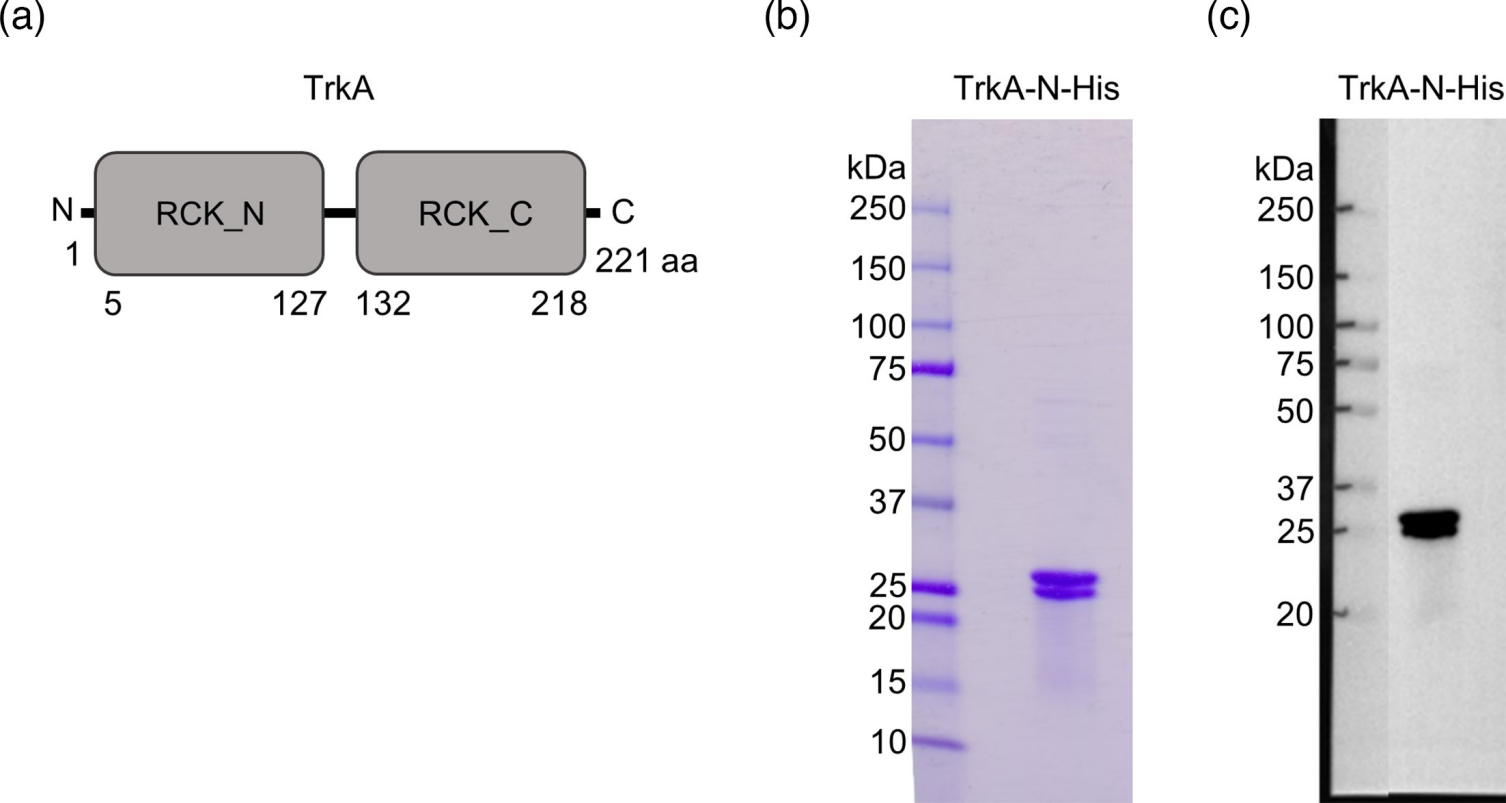
Schematic representation of TrkA domain organization and purified TrkA protein. (**a**) Schematic figure of the full-length TrkA protein domain organization. (**b**) Coomassie-stained SDS-PAGE gel showing recombinant TrkA protein purified with His-tag affinity followed by size exclusion chromatography. (**c**) WB of purified recombinant TrkA detected with *α*His-tag antibody. The recombinant protein had an N-terminal 6× His-tag included in the sequence. N, amino-terminus; C, carboxy-terminus; RCK_N, regulator of K+ conductance domain at the amino-terminus; RCK_C, regulator of K+ conductance domain located at the carboxy-terminus.

Purified recombinant TrkA with an N-terminal 6× His-tag migrated at ~25 kDa in a gradient SDS-PAGE gel (4–20% acrylamide), which corresponds well with the predicted weight of 24.9 kDa for the monomer (Fig. 2b). However, the protein appeared as two separate bands on both the SDS-PAGE gel and the WB (Fig. 2b, c). The polypeptides giving rise to the two observed bands eluted in a single peak during size exclusion chromatography, at a size corresponding to a TrkA octamer (MW ~220 kDa) (Figs S1 and S2). Protein MS analysis identified both the upper and lower bands excised from the SDS-PAGE gel as TrkA. The peptides matching the TrkA aa sequence covered aa 1–113 and 153–205 of the upper band and 1–10, 31–67, 97–118 and 153–205 of the lower band (Fig S3 and Table S5). aa 119–152 and 206–227 were not detected in either band. Since the size of the lower band matched the predicted size of TrkA and peptides matching the same length as the proteins of the two bands were detected, it is unlikely that the size difference is due to the cleavage of the protein in the lower band. Failure to detect a peptide by MS could be due to post-translational modifications to the protein. With the exception of a short peptide (KVLEK), the same peptides were detected in the upper band as in the lower band, and more of the TrkA aa sequence was covered in the upper band than in the lower band, demonstrating that most of the aa sequence of TrkA in the upper band was free of modifications. It is not possible to rule out that TrkA of the upper and lower bands carry different post-translational modifications in the regions corresponding to aa 119–152 and 206–227, but this was not further analysed in this study.

### Recombinant TrkA from *S. mitis* specifically binds c-di-AMP *in vitro*

To determine whether *S. mitis* TrkA binds c-di-AMP, we performed nano differential scanning fluorimetry (nanoDSF) to measure the melting temperature of the protein through intrinsic fluorescence in the absence and presence of various ligands (c-di-AMP, pApA, ATP, AMP and c-di-GMP). NanoDSF experiments showed that the melting temperature (Tm) of TrkA increased with increasing concentration of c-di-AMP, indicating the stabilization of the protein in the presence of ligand and formation of a protein–ligand complex (Fig. S4). Incubation of TrkA in the presence of pApA, AMP, ATP and c-di-GMP showed negligible shifts in the melting temperature at the highest concentration of ligand. Binding affinities (apparent K_d_) were estimated using the FoldAffinity online software [41,42]. The apparent K_d_-values (K_d,app_) indicated that c-di-AMP binding to TrkA is over 1,000-fold greater when compared to the other tested adenine-containing nucleotides, while TrkA binding to c-di-GMP is 100,000-fold lower compared to c-di-AMP (Table 3, Fig. S4). These results show that TrkA specifically binds c-di-AMP *in vitro*.

**Table 3.** Protein–ligand interactions analysed with FoldAffinity online software

Protein	Ligand	Tm±sd (°C)*	T_iso_ (°C)†	K_d,app_ (µM)‡	±sd (µM)
TrkA	–	51.85±0.16	–	–	–
TrkA	c-di-AMP	64.08±0.04	63	0.335	±0.007
TrkA	pApA	52.60±0.07	52	1,650.000	±494.975
TrkA	ATP	52.40±0.07	52	2,610.000	±2390.021
TrkA	AMP	52.35±0.05	54	2,050.000	±494.975
TrkA	c-di-GMP	52.05±0.05	54	100,000.000	±0.000

*Tm represents the melting temperature of the protein at the highest concentration of the respective ligand.

†T_iso_ is the temperature selected for isothermal analysis and plotting of the isothermal fitting curves.

‡K_d,app_ are the apparent binding affinity values at the selected temperatures (T_iso_).

### Creation of *trkA* deletion mutants

The c-di-AMP signalling system affects several phenotypes of *S. mitis* including growth, glucose metabolism, DNA stress tolerance, biofilm formation and acid stress tolerance [16,17]. To determine the potential role of TrkA in these c-di-AMP-regulated phenotypes, in-frame deletion mutants of *trkA* were constructed in the WT background creating the ∆*trkA* mutant and in the Δ*cdaA*, Δ*pde1* and Δ*pde2* mutants giving rise to the Δ*cdaA∆trkA*, Δ*pde1∆trkA* and Δ*pde2∆trkA* double mutants. In addition to this, the *trkA* mutations were complemented by reinserting the *trkA* gene in the original locus and under the natural promoter, creating the KB strains KB*trkA*, Δ*cdaA*KB*trkA*, Δ*pde1*KB*trkA* and Δ*pde2*KB*trkA* (Table 1).

### TrkA does not affect the growth of *S. mitis* in TSB

The Δ*cdaA* mutant grew similarly to the WT, whereas the Δ*pde2* mutant grew slower and reached a lower OD in the stationary phase (Table 4, Fig. S5). The Δ*pde1* mutant displayed a growth pattern similar to WT, but the generation time was 1.27 times longer in this experiment (Table 4). The KB*cdaA* and KB*pde1* strains grew at a similar rate as the WT, whereas the generation time of the KB*pde2* strain was higher than the WT (Table 4). This was as expected from previous publications [16,17]. The generation time of the Δ*trkA* mutant was similar to that of WT, and there was no significant difference in growth rate between the ∆*trkA* mutant and the KB*trkA* strain (Table 4). The *S. mitis* double mutants (Δ*cdaA*Δ*trkA*, Δ*pde1*Δ*trkA* and Δ*pde2*Δ*trkA*) had generation times similar to the respective single mutants (Table 4). Altogether, these results show that *trkA* deletion does not affect the growth of *S. mitis* in TSB.

**Table 4.** Generation time in the exponential phase of growth in TSB

Strain	Generation time (min)±sem†	One-way ANOVA (compared to WT)	One-way ANOVA (compared to the single mutants‡)
WT	37.4±0.4	–	–
Δ*trkA*	39.2±2.9	ns	‡
KB*trkA*	41.6±1.7	*	ns
Δ*cdaA*	38.2±1.3	ns	‡
Δ*cdaA*Δ*trkA*	33.2±1.5	ns	ns
KB*cdaA*	31.7±0.2	ns	*
Δ*cdaA*KB*trkA*	48.1±6.3	****	****
Δ*pde1*	47.6±1.6	***	‡
Δ*pde1*Δ*trkA*	46.2±3.0	**	ns
KB*pde1*	45.0±3.0	*	ns
Δpde1KB*trkA*	48.0±1.8	****	ns
Δ*pde2*	60.0±0.4	****	‡
Δ*pde2*Δ*trkA*	59.7±0.1	****	ns
KB*pde2*	30.4±1.4	*	****
Δ*pde2*KB*trkA*	61.8±0.1	****	ns

*P*-values: *<0.01; **<0.001; ***=0.0001; ****<0.0001; ns>0.05.

†Generation time was calculated in GraphPad with the exponential growth model using only data from the logarithmic phase of growth.

‡Background strains used for comparison to the rest of the mutants within the same group (solid lines define the groups).

###  TrkA and CdaA are required for *S. mitis* growth in low potassium conditions

Considering that c-di-AMP binds to TrkA, and TrkA is important for regulating the intracellular potassium concentration in many bacteria [28,35, 70], we analysed the role of c-di-AMP and TrkA in the growth of *S. mitis* under various potassium concentrations. None of the *S. mitis* strains used in this study were able to grow in potassium-free CDM medium (CDM 0 mM KCl) within the course of the experiment (Fig. S7).

**Fig. 3.**
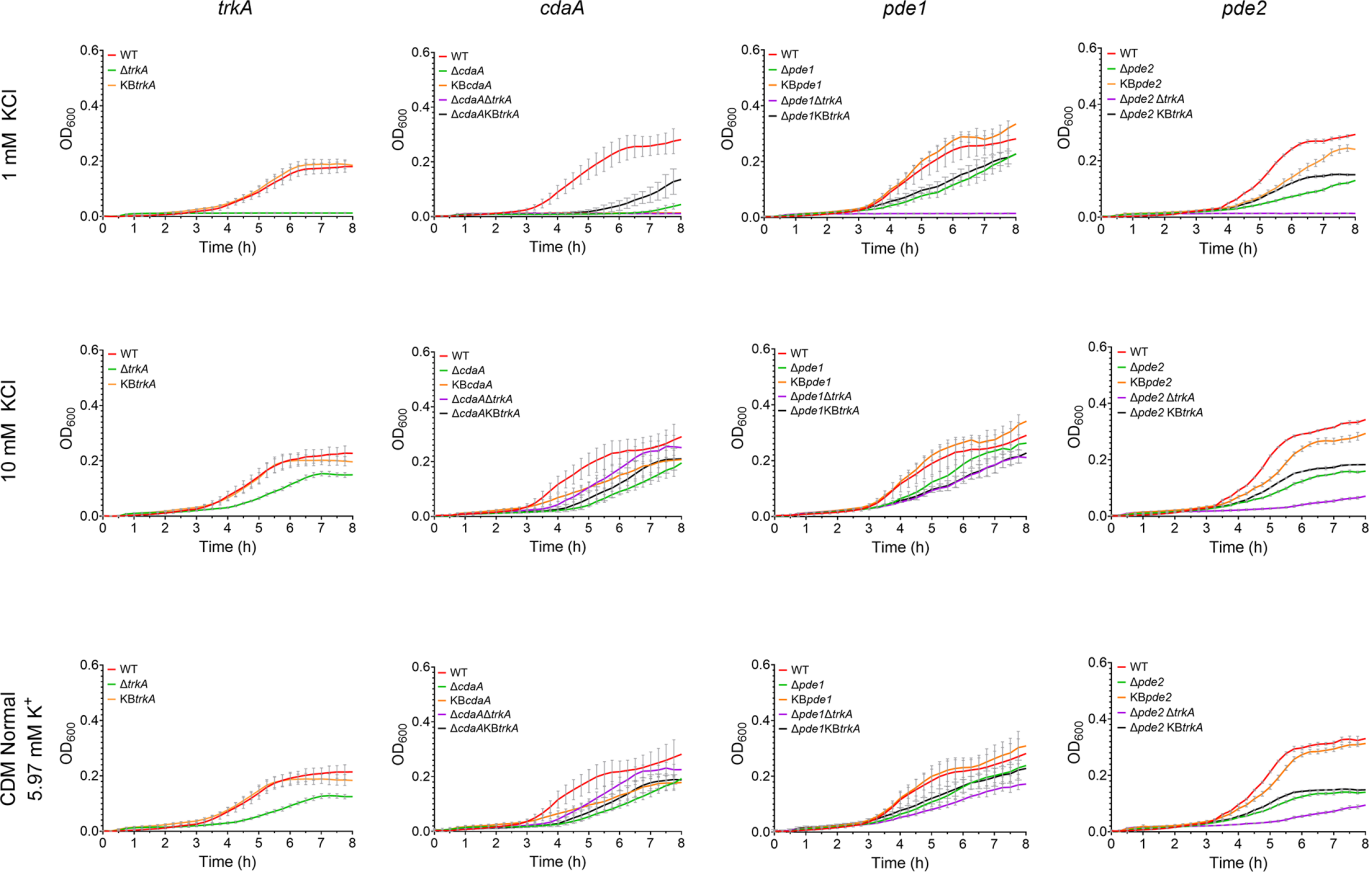
Growth of *S. mitis trkA*,* cdaA*,* pde1*, and *pde2* mutants in CDM with different [K^+^]. The growth curves were plotted using representative data from two independent experiments with two technical replicates in each. All the *S. mitis* strains were grown in CDM medium with either 1 or 10 mM KCl, or in normal CDM medium with 5.97 mM K^+^. Growth of the Δ*cdaA* and Δ*cdaA*Δ*trkA* mutants in CDM with 1 mM KCl was impaired and the two curves overlap.

In CDM supplemented with 1 mM KCl, the mutants of genes encoding c-di-AMP turnover proteins displayed reduced growth compared to the WT (Figs 3a, b, S6, S7a and S7b). The Δ*cdaA* mutant had a longer lag phase compared to the WT, lasting for ~7 h (compared to 3 h for the WT). Re-introduction of *cdaA* into the original genomic location (KB*cdaA* strain) did not rescue the growth of the *cdaA* mutant. Several attempts were made to create a KB*cdaA* strain that restored the growth to WT levels without success (Figs S6 and 7a). The ∆*trkA* single mutant and the ∆*cdaA*∆*trkA*, ∆*pde1*∆*trkA* and ∆*pde2*∆*trkA* mutants were unable to grow in CDM with 1 mM KCl. Growth of the complemented KB*trkA* strains was similar to the respective background strains, meaning the WT strain, ∆*cdaA*, ∆*pde1* or ∆*pde2* mutants, respectively (Figs 3a, b, S7a and S7b).

The Δ*cdaA*, Δ*pde1*, Δ*pde2* and ∆*trkA* mutants grew slower than the WT in CDM containing 10 mM KCl. Whereas the KB*pde1* and KB*trkA* strains grew similarly to the WT, the growth of the KB*pde2* and KB*cdaA* strains was partly restored but not to WT levels. The ∆*pde1∆trkA* and ∆*pde2∆trkA* mutants grew slightly slower than ∆*pde1* and ∆*pde2* mutants, respectively. All strains displayed similar growth in CDM supplemented with 10 mM KCl and standard CDM medium.

Taken together, our results indicate that TrkA is essential for the growth of *S. mitis* under low potassium concentrations and that accurately regulated di-AMP signalling is required for normal growth under these conditions.

### KB*cdaA* strains contain secondary mutations in potassium transport systems

Failure to rescue the growth defect of the *cdaA* mutant in CDM with 1 mM KCl by reintroducing *cdaA* in the original chromosomal location motivated whole-genome sequencing of the ∆*cdaA* mutant and the KB*cdaA* strains.

Sequencing of KB*cdaA* strains created on three separate occasions revealed that all KB*cdaA* strains contained mutations in the TrkH potassium transporter (SM12261_0088; Tables S6 and S7) and that the mutants created on different occasions harboured different mutations in this gene. The KB*cdaA* strain created in our previous study [16] harboured a missense mutation (Gly289Ala) in SM12261_0088 (Table S6). The aa sequence of SM12261_0088 was compared with KtrB of *Vibrio alginolyticus*, which contains four well-conserved glycine residues in the selectivity filter of the cation transporter (Fig. S8). The missense mutation (Gly289Ala; Gly294 in the MSA) in *S. mitis* SM12261_0088 aligns with one of the conserved glycine residues in *V. alginolyticus* KtrB, which has been shown to be essential for high affinity and selective K^+^ uptake [71].

The KB*cdaA* strains created on two different occasions in the current study carried either a nonsense or a frameshift mutation, both leading to the truncation of the cation transporter. The nonsense mutation (268C>T p. Gln90*) resulted in a stop codon at aa residue 90 (aa residue nr 95 in the MSA). The frameshift mutation is a result of a single nucleotide deletion (608delT p. Val203fs; −1 ORF). This shift in the reading frame leads to the introduction of a stop codon at aa position 207 (aa position 212 in the MSA). In addition to mutations in SM12261_0088, mutations present in the KB*cdaA* strains were found in genes putatively involved in DNA replication, transcription, metabolism, synthesis of peptidoglycan, virulence and antibiotic resistance (Table S6). However, the only gene mutated in all KB*cdaA* strains and not mutated in the ∆*cdaA* mutant was *trkH*. Therefore, we hypothesize that secondary mutations in SM12261_0088 explain the inability of the KB*cdaA* strains to grow in 1 mM KCl.

### Quantification of c-di-AMP and expression of *cdaA*,* pde1* and *pde2*

To assess whether intracellular c-di-AMP production and expression of *cdaA*, *pde1* and *pde2* genes were affected in the Δ*trkA* mutant, we grew the WT and mutants under various conditions.

**Fig. 4.**
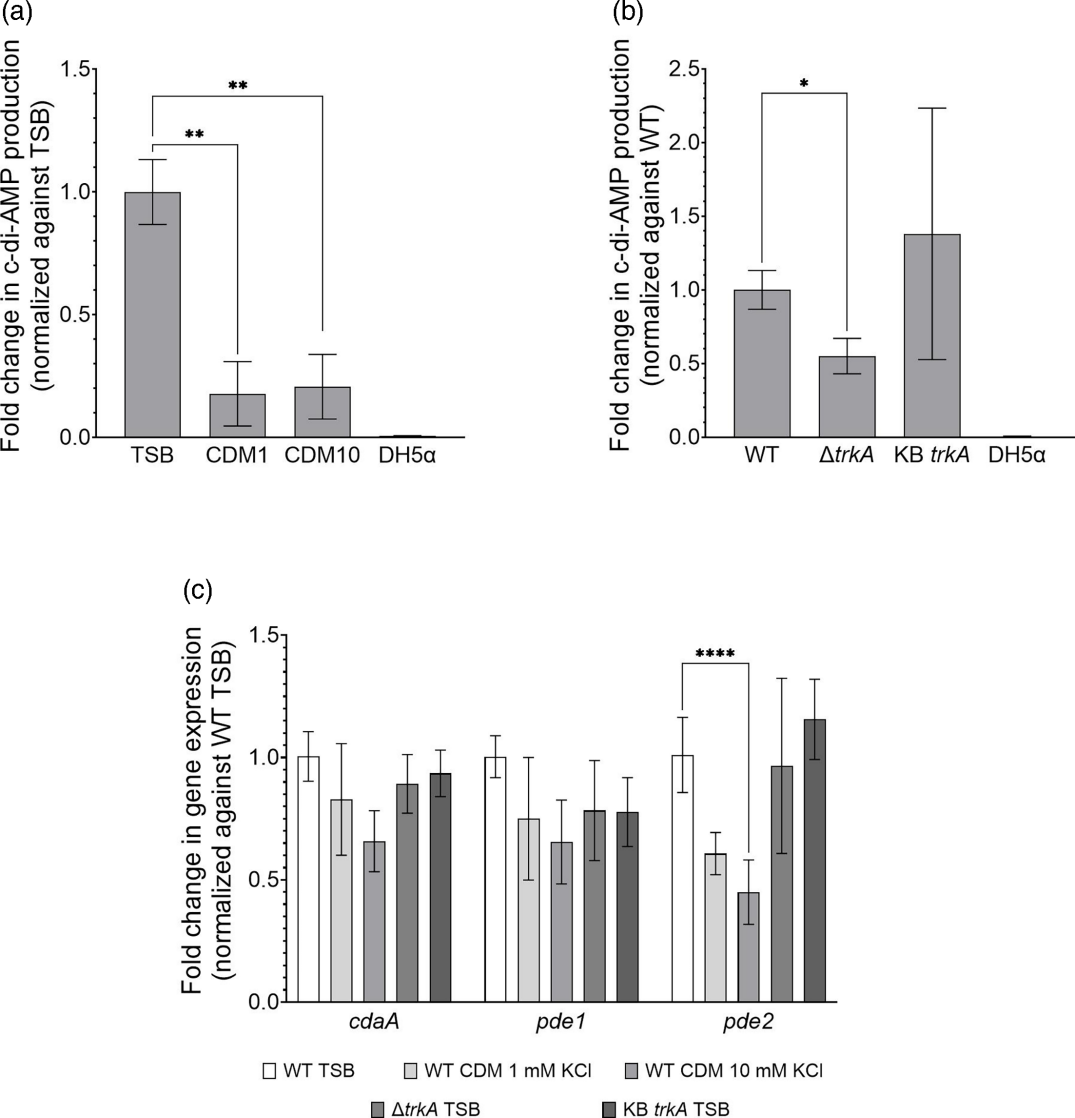
Effect of growth medium, potassium concentration and *trkA* mutation on c-di-AMP concentration and transcription of *cdaA*,* pde1* and *pde2*. C-di-AMP levels measured in bacterial nucleotide extracts with UHPLC-MS/MS. (**a**) C-di-AMP measured in WT incubated in media with different potassium content. (**b**) C-di-AMP measured in strains grown in TSB. *E. coli* DH5*α* was incubated in LB and used as a negative control (the control was below the quantification limit of 3 nM). Statistical significance in graphs (a) and (b) was evaluated with two-tailed unpaired t-test. (**c**) Relative expression of genes encoding c-di-AMP-regulating proteins in different growth conditions. The DNA gyrase A gene (*gyrA*) was used as a reference gene. The relative transcription level of each gene was normalized against WT grown in TSB and calculated by the 2^–ΔΔCt^ method. Statistical analysis was performed with two-way ANOVA followed by Dunnett’s multiple comparison test. Fold change values above 2.0 or below 0.5 were deemed relevant and only the statistics from those were included in the figure. The asterisk indicates statistical significance (*P<0.05; **P<0.002; ****P<0.0001). All graphs show data collected from three independent samples. The bars show average values with sd.

*S. mitis* WT growing in mid-exponential phase had a significantly higher concentration of c-di-AMP when grown in TSB (121±16 ng c-di-AMP/mg protein) than in CDM medium (21±16 in 1 mM KCl and 25±16 ng c-di-AMP/mg protein in 10 mM KCl) (Fig. 4a). The extracellular concentration of potassium in CDM medium did not affect the c-di-AMP concentration. Interestingly, the ∆*trkA* mutant growing in the exponential phase in TSB had a significantly lower intracellular c-di-AMP concentration (67±15 ng c-di-AMP/mg protein; *P*<0.05) compared to the WT (Fig. 4b). The c-di-AMP concentration was restored to WT levels in the KB*trkA* strain (167±104 ng c-di-AMP/mg protein).

Since growth medium and *trkA* deletion affected the c-di-AMP concentration, we analysed the expression level of genes encoding the enzymes regulating the c-di-AMP levels in *S. mitis*. The expression levels of *cdaA*, *pde1* and *pde2* were not affected (less than twofold difference) in the *trkA* mutant compared to the WT in the exponential phase of growth in TSB (Fig. 4c). The expression of *cdaA*, *pde1* and *pde2* was slightly lower in the CDM medium, but the expression was only significantly (twofold) lower for *pde2* in CDM medium containing 10 mM potassium when compared to WT grown in TSB (Fig. 4c). In summary, these results show that the absence of *trkA* significantly reduced intracellular c-di-AMP levels during the logarithmic phase of growth, but there was no direct correlation with the transcription levels of the *cdaA*, *pde1* or *pde2*. Furthermore, the extracellular potassium concentration did not impact c-di-AMP production under the conditions tested in this study.

### TrkA is not involved in tolerance of *S. mitis* to NaCl-mediated osmotic stress

**Fig. 5.**
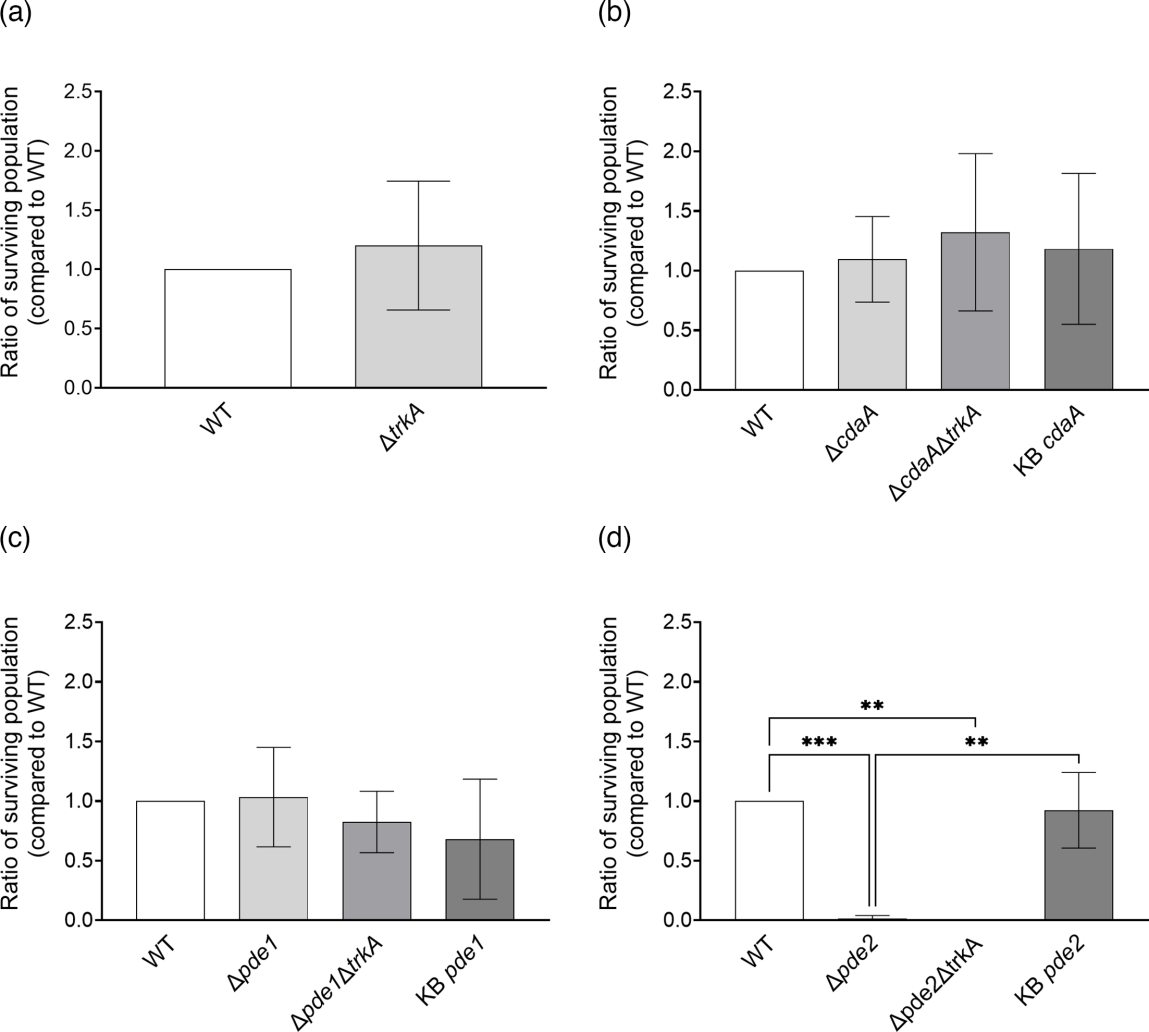
Osmotic stress tolerance in *S. mitis trkA* deletion mutants. Comparison of bacterial populations surviving osmotic stress (0.2 M NaCl) in (**a**) WT background, (**b**) Δ*cdaA* background, (**c**) Δ*pde1* background and (**d**) Δ*pde2* background. Unpaired two-tailed t-test was used to calculate statistical significance in figure (a) and one-way ANOVA followed by Šidák’s multiple comparison test for figures (b)–(d). The asterisks indicate statistical significance (**P<0.009; ***P<0.0003). Error bars represent sd.

*S. mitis* strains were exposed to osmotic stress in the form of 0.2 M NaCl. The ∆*cdaA* and ∆*pde1* mutants did not display altered osmotic stress tolerance compared to the WT (Fig. 5). The ∆*pde2* mutant on the other hand was substantially impaired in growth under osmotic stress compared to the WT, and this was restored to WT level in the KB*pde2* strain. There was no difference in growth under osmotic stress between the ∆*trkA* mutant and the WT. There was also no difference between the ∆*cdaA*∆*trkA*, ∆*pde1*∆*trkA* or ∆*pde2*∆*trkA* mutants compared to the ∆*cdaA*, ∆*pde1* and ∆*pde2* single mutants*,* respectively. This shows that the c-di-AMP signalling network is involved in regulating osmotic stress tolerance of *S. mitis*, but TrkA is not an essential effector protein under the conditions tested in this study.

### TrkA is involved in UV stress tolerance of *S. mitis*

**Fig. 6.**
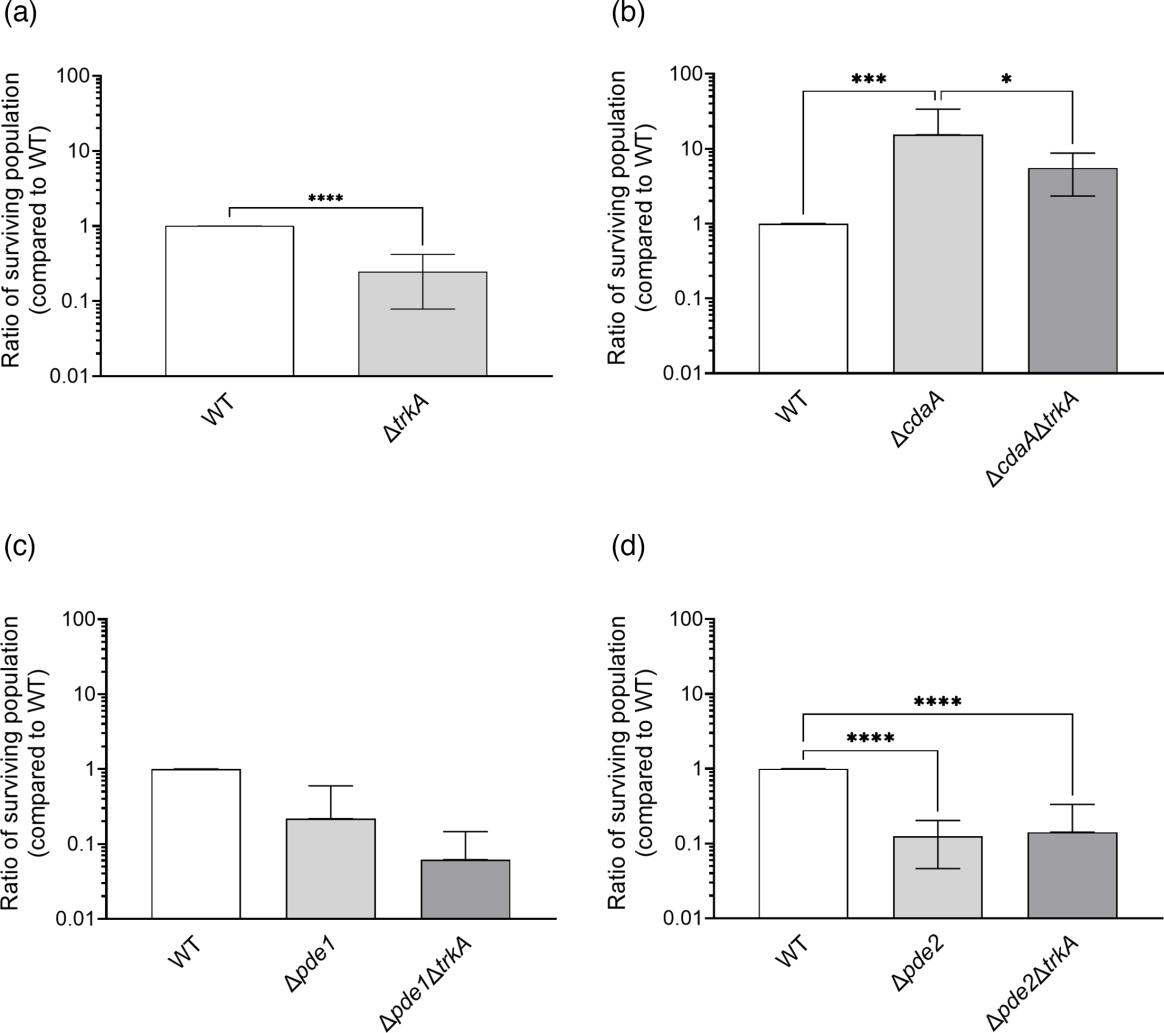
UV stress tolerance in *S. mitis trkA* deletion mutants. Comparison of the influence of *trkA* in bacterial populations surviving UV radiation (2.5 mJ/cm^2^) in (**a**) WT background, (**b**) Δ*cdaA* background, (**c**) Δ*pde1* background and (**d**) Δ*pde2* background. Unpaired two-tailed t-test was used to calculate statistical significance in figure (a) and one-way ANOVA followed by Šidák’s multiple comparison test for figures (b)–(d). The asterisks indicate statistical significance (*P<0.05; ***P<0.0002; ****P<0.0001). Error bars represent 95% CIs.

The disruption of the c-di-AMP signalling system alters the DNA stress tolerance of *S. mitis* [17]. Similar to the previously published data, the *cdaA* mutant survived exposure to UV radiation better than the WT, and the *pde1* and *pde2* mutants had a decreased ability to survive UV radiation compared to the WT (Fig. 6). The Δ*trkA* mutant displayed significantly lower survival compared to the WT when exposed to a dose of 2.5 mJ cm^2^ (Fig. 6a). The Δ*cdaA* mutant survived UV stress better than the Δ*cdaA*Δ*trkA* mutant (Fig. 6b). However, there was no significant difference in UV stress survival between the ∆*pde1* and ∆*pde1trkA* mutants (Fig. 6c) or ∆*pde2* and ∆*pde2trkA* mutants (Fig. 6d). This may suggest that TrkA is involved in the UV stress response in *S. mitis*.

### TrkA is not involved in DNA stress tolerance inferred by ciprofloxacin

DNA stress tolerance was also tested using the antibiotic ciprofloxacin (Table 5). The ∆*cdaA* mutant was more tolerant, and the ∆*pde1* and ∆*pde2* mutants were less tolerant to ciprofloxacin compared to the WT, which corresponded well with our previously published data [17]. The deletion of *trkA* alone or in the background of the c-di-AMP-regulating gene mutants did not affect the strains’ susceptibility to these antibiotics compared to the respective single mutants. The only exception is the Δ*pde2*Δ*trkA* double mutant, which had an increased tolerance to ciprofloxacin compared to the Δ*pde2 *mutant. However, the Δ*pde2*KB*trkA* strain displayed similar tolerance to ciprofloxacin as the Δ*pde2*Δ*trkA* double mutant, which indicates that the marginally increased tolerance was not due to the *trkA* mutation. This suggests that TrkA is not involved in a c-di-AMP-regulated DNA stress response against ciprofloxacin.

**Table 5.** MICs of antibacterial compounds

	Antibacterial compound (mg l^−1^)	
	**AMP (0.0039–0.0625**)	**CIP (0.25–8**)
WT	0.0625	2
Δ*trkA*	0.0625	2
Δ*cdaA*	0.0625	4
Δ*cdaA*Δ*trkA*	0.0625	4
Δ*pde1*	0.0625	1
Δ*pde1*Δ*trkA*	0.0625	1
Δ*pde2*	0.0625	1
Δ*pde2*Δ*trkA*	0.0625	2
Δ*pde2*KB*trkA*	0.0625	2

Numbers in the parentheses indicate the concentration range used in the experiment.

AMP, ampicillin; CIP, ciprofloxacin.

### TrkA is not involved in ampicillin tolerance of *S. mitis*

We previously showed that the ∆*cdaA* and ∆*pde2* mutants had a slightly lower tolerance (twofold difference) to the cell wall-disrupting antibiotic ampicillin compared to the WT, whereas the *pde1* mutant displayed a similar MIC as the WT [17]. Here, we show that the ∆*cdaA*, ∆*pde1* and ∆*pde2* mutants display similar MIC of ampicillin (Table 5). The deletion of *trkA* did not affect the ampicillin tolerance of the WT or the ∆*cdaA*, ∆*pde1* or ∆*pde2* mutants. These results show that TrkA is not involved in tolerance of *S. mitis* to the cell wall-disrupting antibiotic ampicillin.

### TrkA is not a major c-di-AMP-regulated effector protein in biofilm formation

**Fig. 7.**
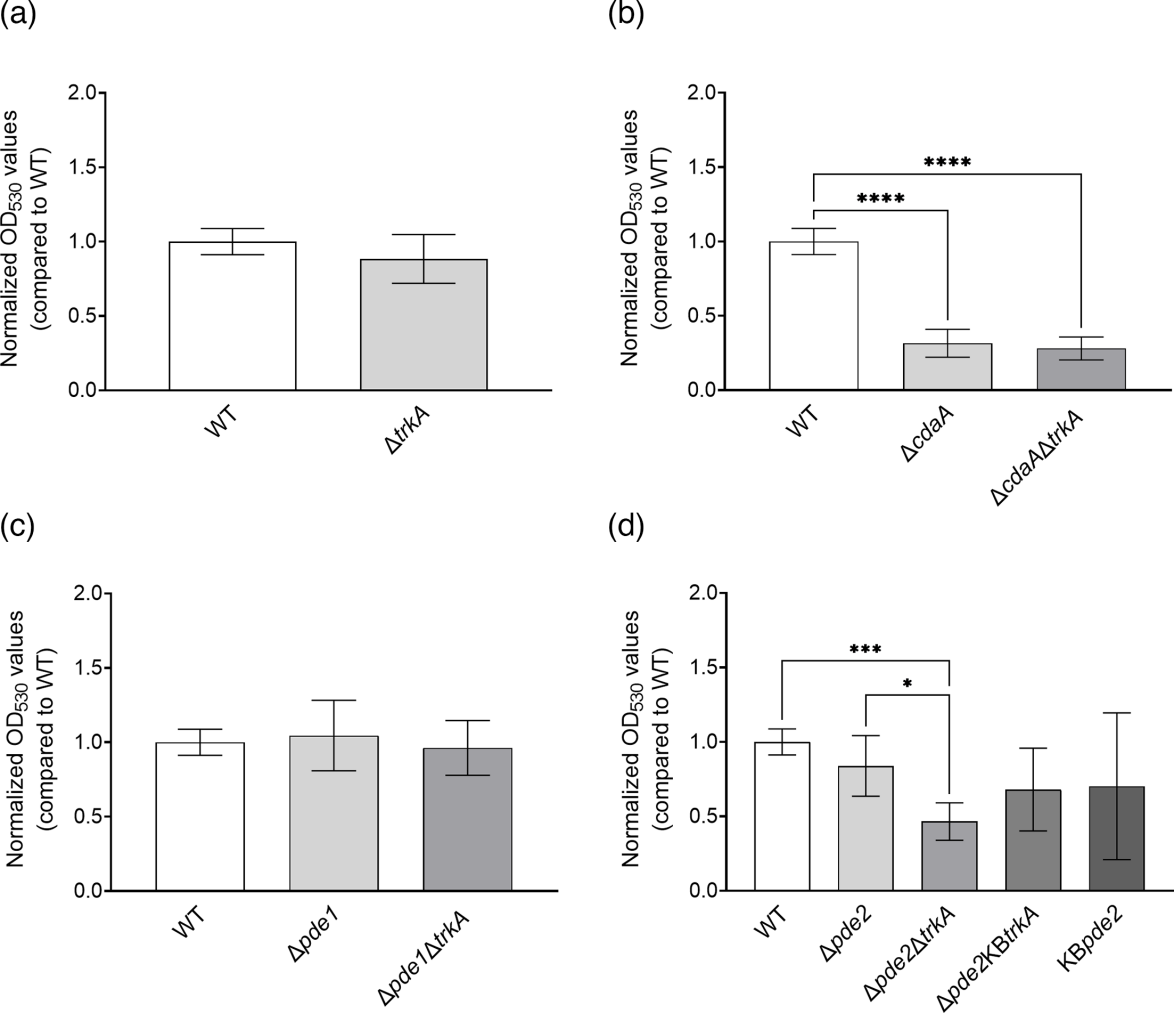
The role of TrkA in c-di-AMP-mediated biofilm formation. Comparison of the effect of *trkA* mutation on bacterial biofilm formation in (**a**) WT background, (**b**) Δ*cdaA* background, (**c**) Δ*pde1* background and (**d**) Δ*pde2* background. Statistical significance was calculated with an unpaired two-tailed t-test in figure (a) and one-way ANOVA followed by Šidák’s multiple comparison test for figures (b)–(d). The asterisks indicate statistical significance (*P<0.03; ***P<0.0002; ****P<0.0001). Error bars represent 95% CIs.

Biofilm formation of *S. mitis* is regulated by c-di-AMP [17], but the effector proteins of the signalling cascade have not yet been identified. We therefore investigated the role of TrkA in biofilm formation in TSB medium and a 5% CO_2_ atmosphere. Among single mutants of genes encoding c-di-AMP-regulating proteins, only the Δ*cdaA* mutant displayed altered biofilm formation compared to the WT (Fig. 7b), which is in agreement with our previous study [17]. The *trkA* mutant did not display altered biofilm formation compared to the WT (Fig. 7a). Similarly, the deletion of *trkA* did not alter the biofilm-forming capability of the ∆*cdaA∆trkA* and ∆*pde1∆trkA* mutants compared to the respective ∆*cdaA* and ∆*pde1* single mutants. However, *trkA* deletion in the background of the Δ*pde2* mutant resulted in significantly decreased biofilm formation in the double mutant, compared to the single mutant and the WT strain. However, the ∆*pde2*KB*trkA* strain formed a biofilm mass intermediary of the ∆*pde2* mutant and the ∆*pde2*∆*trkA* mutant but not statistically significantly different from either strain. These results show that TrkA is not a major effector protein in the c-di-AMP-regulated biofilm formation.

## Discussion

In this study, we characterize an RCK domain protein, TrkA. RCK domain proteins have been shown to bind c-di-AMP and other adenine-containing nucleotides and are known regulators of potassium channels and transporters in both prokaryotic and eukaryotic cells [28,65, 72,77]. *S. mitis* TrkA is a homologue of CabP from *S. pneumoniae*; Trk1 of *Streptococcus mutans*; KtrA from *S. agalactiae*, *S. aureus* and *Bacillus subtilis*; and KtrC of *B. subtilis* that have been shown to bind c-di-AMP. In agreement with this, TrkA from *S. mitis* binds specifically to c-di-AMP at an affinity similar to CabP of *S. pneumoniae*, and KtrA of *S. aureus*, but an order of magnitude lower than for KtrA of *B. subtilis* and Trk1 of *S. mutans* and an order of magnitude higher than KtrC of *B. subtilis* [25,69, 75, 78]. Since the apparent K_d_ of TrkA from *S. mitis* is similar to the K_d_ of the almost identical protein CbpP of *S. pneumoniae*, it is unlikely that the two bands detected in our SDS-PAGE analysis had a significant effect on the affinity of TrkA for c-di-AMP.

**Fig. 8.**
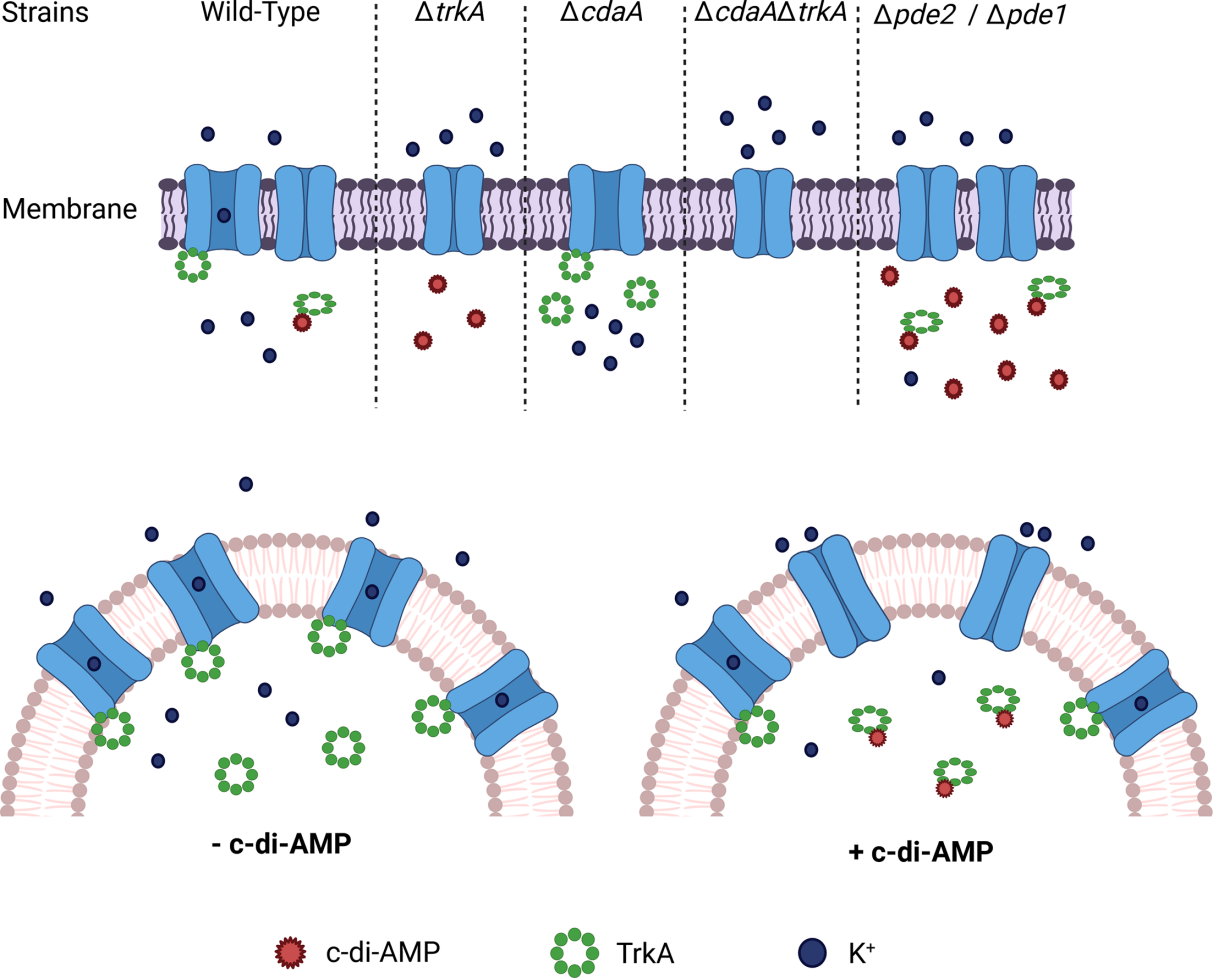
Working model for c-di-AMP-mediated regulation of TrkA and potassium uptake in *S. mitis*. TrkA associates with the membrane-embedded TrkH-type K^+^ transporter, opening the channel for K^+^ import. When c-di-AMP binds to TrkA, it dissociates from the K^+^ transporter and represses K^+^ import. The absence of c-di-AMP (Δ*cdaA*) results in an unregulated open configuration of the K^+^ transporter, allowing for uncontrolled K^+^ transport. High c-di-AMP concentration (Δ*pde1* or Δ*pde2*) results in reduced interaction between TrkA and the K^+^ transporter, closing the channel and repressing K^+^ uptake. The absence of TrkA (Δ*trkA*, Δ*cdaA*Δ*trkA*, Δ*pde1*Δ*trkA* and Δ*pde2*Δ*trkA*) results in a constitutively closed configuration of the K^+^ transporter and absence of K^+^ import through this transporter. The model for c-di-AMP-mediated regulation of potassium transport in TrkA of *S. mitis* is based on, and our data supports, the previously published model of c-di-AMP-regulated potassium uptake by the TrkA and TrkH orthologues in *S. pneumoniae* introduced by Bai *et al*. [25]. Created in BioRender (https://BioRender.com/h22w343).

Potassium transport is highly regulated, and c-di-AMP is crucial for transcriptional and allosteric regulation of potassium transporter systems in many bacteria [25,27,29, 79]. We show that TrkA is essential for the growth of *S. mitis* under low potassium conditions (1 mM) but dispensable for growth at higher concentrations (10 mM). Similar results were described for CabP in *S. pneumoniae* D39, KtrC of *B. subtilis* and KtrA of *S. aureus* [25,69, 80]. TrkH-like potassium transporters are functional when the membrane-bound channel interacts with the cytosolic regulator. C-di-AMP negatively regulates potassium import, and it has been shown that c-di-AMP binding to the regulator CabP reduces the interaction between CabP and the potassium transporter [25]. We have previously shown that c-di-AMP levels are undetectable in the *S. mitis* ∆*cdaA* mutant and increased in the ∆*pde1* and ∆*pde2* mutants. We show in this study that the ∆*cdaA* mutant displays a severely reduced growth rate at low extracellular potassium concentration, whereas the ∆*pde1* and ∆*pde2* mutants display slightly reduced growth in CDM but grow equally well at 1 mM and 10 mM potassium. A working model for the c-di-AMP-mediated regulation of Trk-like potassium transporters was introduced by Bai *et al*. [25]. This model proposes that at low c-di-AMP levels, the TrkA-type regulator is associated with the transporter which is open, whereas at high c-di-AMP levels, the TrkA-type regulator dissociates from the transporter and the transporter closes (Fig. 8). The conformation of the transporter is closely regulated by c-di-AMP and the activity of c-di-AMP turnover proteins. Based on this model, the potassium channel is unregulated and continuously open in the ∆*cdaA* mutant, which leads to uncontrollable potassium influx, resulting in potassium toxicity as suggested by Gundlach *et al*. [70]. The moderate effect on growth in the low potassium environment of the ∆*pde1* and ∆*pde2* mutants was unexpected since increased c-di-AMP levels should dissociate TrkA from the transporter, inhibit potassium import and affect growth negatively as shown for a Δ*pde1*Δ*pde2* double mutant in *S. pneumoniae* D39 [25,81]. However, the c-di-AMP concentration measured for the WT is relatively low in the CDM medium independent of the potassium concentration, and it is possible that the c-di-AMP levels do not reach the critical level to completely dissociate TrkA from the transporter, enabling regulated potassium import in the ∆*pde1* and ∆*pde2* mutants.

The absence of TrkA did not significantly affect the growth of *S. mitis* in rich medium, and the growth of the ∆*trkA* mutant was restored to WT levels in CDM medium at higher potassium concentrations, demonstrating that TrkA is not essential for growth in potassium-rich environments. The dispensability of TrkA in *S. mitis* under these conditions might be explained by multiple putative potassium transport systems that are active under different conditions as demonstrated for other bacteria [80,82]. Three putative c-di-AMP-regulated potassium transport systems have been identified in *S. mitis* [34], and additional experiments are required to understand their role and hierarchy in potassium homeostasis.

The re-introduction of *cdaA* in the ∆*cdaA* mutant strain did not restore the growth under low potassium conditions. Whole-genome sequencing identified secondary mutations in the Δ*cdaA* and KB*cdaA* strains. The KB*cdaA* strains specifically had mutations in the gene coding for the potassium transporter protein TrkH, which is part of the *trkA/trkH* operon. TrkH is likely the permease that TrkA interacts with and regulates in response to the c-di-AMP levels as shown in *S. pneumoniae* [25], and secondary mutations in *trkH* can explain the inability of the KB*cdaA* strain to grow in low potassium conditions. This is in line with studies on *S. agalactiae* and *B. subtilis*, which have reported that the disruption of the c-di-AMP signalling system is associated with secondary mutations affecting cation homeostasis and osmoregulation [31,70]. We have previously shown that the re-introduction of *cdaA* complements the ∆*cdaA* mutation with regard to intracellular c-di-AMP concentration, static growth in TSB, chain length [16] and biofilm formation [17], demonstrating that the secondary mutations in *trkH* do not affect these phenotypes.

It has previously been shown that the intracellular c-di-AMP levels are downregulated at low environmental potassium concentration [27,35]. In *B. subtilis,* this is achieved by downregulated expression of the diadenylate cyclase CdaA [70], whereas in *S. pneumoniae*, the c-di-AMP level is regulated post-translationally. In contrast to these results, the c-di-AMP concentration was similar in *S. mitis* grown in CDM containing 1 and 10 mM potassium, indicating that the extracellular potassium concentration did not influence c-di-AMP homeostasis in *S. mitis* under the conditions tested. In *S. pneumoniae*, the intracellular c-di-AMP concentration is downregulated in a mutant of the TrkA homologue CabP [35]. We consistently detected significantly lower intracellular c-di-AMP levels in the ∆*trkA* mutant compared to the WT. The transcription of *cdaA*, *pde1* and *pde2* remained unchanged, indicating potential post-transcriptional regulation of the c-di-AMP concentration. Similar results were reported for the ∆*cabP* mutant in *S. pneumoniae*, but direct interaction between CabP and CdaA was not detected [35], and the mechanism for a TrkA-dependent regulation of c-di-AMP concentration is currently not known.

TrkA homologues and potassium homeostasis are involved in c-di-AMP-mediated phenotypes in diverse bacteria [63,78, 83,85]. Since, in *S. mitis*, c-di-AMP is involved in regulating growth in TSB, colony morphology, chain length, DNA stress tolerance, biofilm formation [16,17], growth under low potassium conditions and osmotic stress (this study), we investigated the function of TrkA as a c-di-AMP-regulated effector protein in these processes. The *S. mitis* ∆*pde2* mutant was more susceptible to hyperosmotic stress compared to the WT, indicating that c-di-AMP is an important regulator of osmotic stress tolerance in *S. mitis*. Interestingly, the deletion of *pde2*, but not *pde1,* resulted in reduced hyperosmotic stress tolerance in *S. mitis*. This is in contrast to data presented for *S. pneumoniae*, where the ∆*pde1* mutant was more susceptible to hyperosmotic stress than the ∆*pde2* mutant [35]. Several other studies have shown that mutants of GdpP-type phosphodiesterase encoding genes are more susceptible to hyperosmotic stress compared to the WT [7,86]. The reason for these contrasting results in *S. mitis* and other bacteria is not known and will be addressed in future studies. TrkA is not involved in hyperosmotic stress tolerance under the conditions tested. This is in contrast to the Δ*ktrC* mutant of *S. aureus*, which displayed significant hyperosmotic stress defects in rich medium [87]. However, several studies have shown that inactivation of TrkA homologues only results in increased susceptibility to hyperosmotic stress under low potassium conditions [26,82, 88], whereas our experiments were performed in rich medium with standard potassium concentration. The *S. mitis* Δ*trkA* and Δ*cdaA*Δ*trkA* mutants are more susceptible to UV stress than their respective background strains. The role of TrkA in UV stress tolerance is currently not known, and it is possible that the effect can be attributed to failure to maintain potassium homeostasis required for the activation of UV-induced stress responses. We did not detect a role for TrkA in growth in TSB, colony morphology, chain length or biofilm formation.

In conclusion, TrkA from *S. mitis* is a c-di-AMP-binding effector protein that is essential for the regulation of potassium homeostasis under low environmental potassium conditions but appears to be dispensable at higher environmental potassium concentrations. According to the working model based on published data from other bacteria and data generated in this study, TrkA is a cytosolic regulator of the membrane-integrated potassium importer TrkH. Combining data from available studies, the model suggests that in the absence of c-di-AMP, TrkA is bound to TrkH, which is open, and potassium is imported through the transporter. Upon binding of c-di-AMP, TrkA dissociates from TrkH and closes the transporter, which reduces import of potassium (Fig. 8). TrkA may have a role in UV stress tolerance, but further experiments are needed to understand the potential mechanism. TrkA does not seem to be involved in any other c-di-AMP-mediated phenotypes identified so far in *S. mitis*, and future experiments are required to identify the effector proteins involved in growth in TSB, chain length regulation, biofilm formation, ciprofloxacin tolerance and survival in a hyperosmotic environment.

## Supplementary material

10.1099/mic.0.001597 Uncited Supplementary Material 1.

## Abbreviations

AA: amino acid
c-di-AMP: cyclic di-adenosine monophosphate
CDM: chemically defined medium
KB: knock-back
LB: lysogeny broth
MSA: multiple sequence alignment
MW: molecular weight
nanoDSF: nano differential scanning fluorimetry
RT: room temperature
TSB: tryptone soy broth
UHPLC-MS/MS: ultra-high-performance liquid chromatography coupled to tandem mass spectrometry
WB: Western blot
