# Supplementary material for: TrkA of Streptococcus mitis CCUG31611 binds cyclic di-adenosine monophosphate and is required for growth in low potassium conditions
Source: Microbiology (Reading). 2025 Aug 14;171(8):001597. doi: 10.1099/mic.0.001597 (PMC12453120; doi:10.1099/mic.0.001597)
Supplement: Uncited Supplementary Material 1. [file mic-171-01597-s001.pdf]

SUPPLEMENTARY DATA

Table S1. Pairwise comparison of RCK domains to *S. mitis* TrkA.

Strain name	Locus name	RCK_N % ID	RCK_C % ID
***Streptococcus* mitis CCUG31611**	SM12261_0089 **versus**	-	-
***Streptococcus agalactiae* NEM316**	GBS1678	52.1	32.1
***Streptococcus gallolyticus* UCN34**	Gallo_1832	48.7	34.5
***Streptococcus mutans* UA159**	SMU_1562	25.4	20.7
***Staphylococcus aureus* subsp. Aureus USA300_FPR3757**	SAUSA300_0988	41.2	29.5
***Streptococcus pneumoniae* D39**	SPD_0077	96.6	86.9
**Strain name**	**Protein / Protein domain**	**RCK_N % ID**	**RCK_C % ID**
**Bacillus subtilis strain 168**	KtrA	33.6	21.8
**Staphylococcus aureus 08BA02176**	KtrA RCK_C domain	-	24.5

Table S2. Primers used it this study.

**CONSTRUCTION OF MUTANTS**		
**Primer name**	**Primer sequence (5'-3')**	**Primer function**
**RS252**	TGTTGTTGCTGGAGCAGG	Amplification of the upstream *trkA* gene region in *S. mitis* CCUG 31611
**RS254**	CCCCTCCTTACATCATTCTTCCTTTC TTATC	Amplification of the upstream *trkA* gene region in *S. mitis* CCUG 31611
**RS255**	AGAATGATGTAAGGAGGGGGATCC CC	Amplification of the downstream *trkA* gene region in *S. mitis* CCUG 31611
**RS256**	TGCCAAGTCTACTTCGTAG	Amplification of the downstream *trkA* gene region in *S. mitis* CCUG 31611
**RS258**	ATGTTGTTGGGAATTACAGC	Screening for Δ*trkA* or *trkA*
**RS259**	CAGAAGCCTCCGCCAAG	Screening for Δ*trkA* or *trkA*
**GHR17**	ACAGGACCACCACCTACAAT	Screening for Δ*cdaA* or *cdaA*
**GHR18**	CAAGGTCACACGATTAGCTC	Screening for Δ*cdaA* or *cdaA*
**GHR19**	CCAATGTGATTTATCGTCGT	Screening for Δ*pde1* or *pde1*
**GHR20**	TTAATTGCTTTTGCTTCTGC	Screening for Δ*pde1* or *pde1*
**GHR21**	ATCAACATCAACATCCAAGC	Screening for Δ*pde2* or *pde2*
**GHR22**	ACGGATCAATGGGTAAGTTT	Screening for Δ*pde2* or *pde2*
**FP1242**	CTTGAGCTGGGCTTCGTAGT	Amplification of Erythromycin cassette
**FP1243**	ACAGGGGATGTCATGGGTAA	Amplification of Erythromycin cassette

(Continued on the next page)

GENE CLONING		
**Primer name**	**Primer sequence (5'-3')**	**Primer function**
**RS450**	GATCCATATGCACCACCATCATCAC CATTCAGATCGTACGATTGG	Amplification of *trkA* coding sequence
**RS435**	GATCAAGCTTTTACGAATTTAATTCT GCCAAA	Amplification of *trkA* coding sequence
**RS164**	ACGATGCGTCCGGCGTAG	Screening for *trkA* cloning sequence in the pET30b_TrkA-N-His plasmid
**RS163**	TCAGCAAAAAACCCCTCAAGAC	Screening for *trkA* cloning sequence in the pET30b_TrkA-N-His plasmid

Table S3. Composition of the modified Chemically Defined Medium (CDM).

**Component type and name**	**Vendor / Distributor**	**Final concentration**
**SALTS**		
**Iron (II) sulfate heptahydrate**	Merck	5,04 mg/L
**Iron (III) nitrate nonahydrate**	VWR	1.06 mg/L
**Dipotassium hydrogen phosphate***	Sigma	0 or 200 mg/L
**Potassium dihydrogen phosphate***	Sigma	0 or 500 mg/L
**Magnesium sulfate heptahydrate**	Millipore	700 mg/L
**Manganese (II) sulphate**	Sigma	5,58 mg/L
**Calcium chloride dihydrate**	Millipore	20 mg/L
**Sodium acetate trihydrate**	SIGALD	2 720 mg/L
**Sodium bicarbonate**	Sigma	2 500 mg/L
**Sodium phosphate monobasic monohydrate**	Sigma	3 200 mg/L
**Disodium hydrogen phosphate dihydrate**	Millipore	9 220 mg/L
**Potassium chloride***	Sigma	0; 1.11; 11.11 mM
**AMINO ACIDS**		
**L-Alanine**	Sigma	100 mg/L

(Continued on the next page)

**L-Arginine**	Sigma	100 mg/L
**L-Aspartic acid**	Sigma	100 mg/L
**L-Asparagine**	Sigma	100 mg/L
**L-Cysteine**	Sigma	500 mg/L
**L-Cystine**	Sigma	50 mg/L
**L-Glutamic acid**	Sigma	100 mg/L
**L-Glutamine**	Sigma	200 mg/L
**Glycine**	Sigma	100 mg/L
**L-Histidine**	Sigma	100 mg/L
**AMINO ACIDS**		
**L-Isoleucine**	Sigma	100 mg/L
**L-Leucine**	Sigma	100 mg/L
**L-Lysine**	Sigma	100 mg/L
**L-Methionine**	Sigma	100 mg/L
**L-Phenyalanine**	Sigma	100 mg/L
**L-Proline**	Sigma	100 mg/L
**Hydroxy-L-Proline**	Aldrich	100 mg/L
**L-Serine**	Aldrich	100 mg/L
**L-Threonine**	Sigma-Aldrich	200 mg/L
**L-Tryptophane**	Sigma	100 mg/L
**L-Tyrosine**	Sigma	100 mg/L
**L-Valine**	Sigma-Aldrich	100 mg/L
**VITAMINS**		
**p-Aminobenzoic acid**	Sigma	0,2 mg/L
**Biotin**	Sigma	0,2 mg/L

(Continued on the next page)

**Folic acid**	Sigma	0,8 mg/L
**Niacinamide**	Sigma	1 mg/L
**β-Nicotinamide adenine dinucleotide**	Merck	2,5 mg/L
**Pantothenate calcium salt**	Sigma	2 mg/L
**Pyridoxal**	Merck	1 mg/L
**Pyridoxamine**	Merck	1 mg/L
**(-)-Riboflavin**	Sigma	1,6 mg/L
**Thiamine**	Merck	1 mg/L
**Vitamin B12**	Sigma	0,1 mg/L
**VITAMINS**		
**Adenine**	Sigma	20 mg/L
**Guanine**	Sigma	20 mg/L
**Uracil**	Sigma	20 mg/L
**OTHER**		
**Choline**	Sigma	20 mg/L
**D-(+)-Glucose**	Sigma	10 000 mg/L

*The concentrations of this compound were adjusted dependent on which variant of CDM medium was made (see Table S3).

Table S4. Content of potassium compounds in each CDM medium variant.

	**Adjusted compounds**			**Total concentration (mM)**
**CDM medium name**	**KCl**	**K_2HPO_4**	**KH_2PO_4**	**$[K^+]$**
**CDM 0 mM KCl**	0 mM	0 mM	0 mM	0 mM
**CDM 1.11 mM KCl**	1.11 mM	0 mM	0 mM	1.11 mM
**CDM 11.11 mM KCl**	11.11 mM	0 mM	0 mM	11.11 mM
**Normal CDM**	0 mM	1.15 mM	3.67 mM	5.97 mM

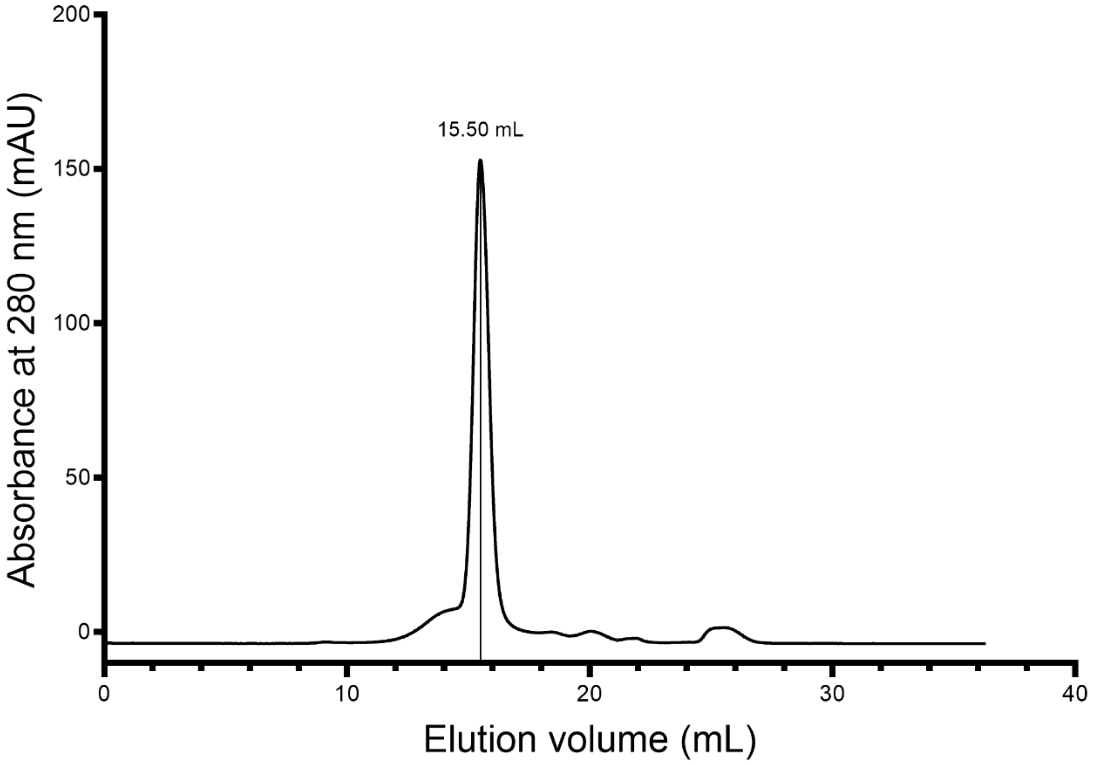

Figure S1. Size exclusion chromatography of TrkA recombinant protein. A Sepharose 6 Increase 10/300 GL column was used. The x-axis of the chromatogram shows the elution volume in milliliters (mL) and the y-axis shows the absorbance measured at 280 nm in milli-absorbance units (mAU).

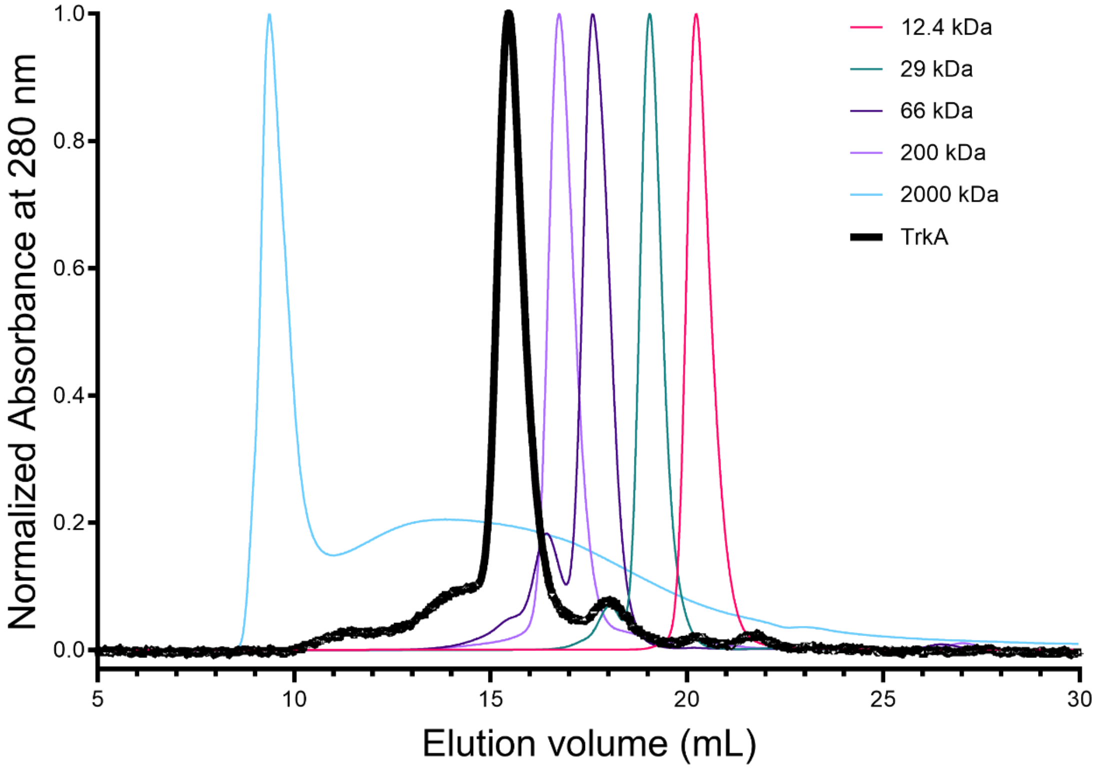

Figure S2. TrkA forms multimer in solution. The elution profile of the recombinant TrkA shown in comparison to various proteins of known molecular weights run under the same conditions. The theoretical size of the TrkA monomer is ≈25 kDa. The protein standard included cytochrome c (12.4 kDa), carbonic anhydrase (29 kDa), bovine serum albumin (66 kDa), β-amylase (200 kDa) and blue dextran (2000 kDa).

High TrkA band:

MHHHHHHSDRTIGILGLGIFGSSVLTALAKQDMNIIAIDDHAERINQFEPVLARGVVGDITDEELLRTAGIDTC
DTVVVATGENLESSVLAVMHCKSLGVPRVIAKVKSQTAKKVLEKIGADSVISPEYEMGQSLAQTILFHNNVD
VFQLDKNVSIVEMKIPSVWAGQSLSQLDLRGKYNLNVLGFREQENSPLDVQFGPNDLLRSDAYIMAVINNQY
LDDLAELNS-

Low TrkA band:

MHHHHHHSDRTIGILGLGIFGSSVLTALAKQDMNIIAIDDHAERINQFEPVLARGVVGDITDEELLRTAGIDTC
DTVVVATGENLESSVLAVMHCKSLGVPRVIAKVKSQTAKKVLEKIGADSVISPEYEMGQSLAQTILFHNNVD
VFQLDKNVSIVEMKIPSVWAGQSLSQLDLRGKYNLNVLGFREQENSPLDVQFGPNDLLRSDAYIMAVINNQY
LDDLAELNS-

Figure S3. TrkA samples analyzed with mass spectrometry. The two bands, high and low, indicate the protein bands excised from the SDS-PAGE gel presented in Fig. 2B. The amino acids detected in the *S. mitis* TrkA protein sequence are marked in yellow.

Table S5. Peptides detected in TrkA protein bands with mass spectrometry.

Peptide sequence	High band	Low band
**EQENSPLDVQFGPNDLLR**	Detected	Detected
**GKYNLNVLGFR**	Detected	Detected
**GKYNLNVLGFREQENSPLDVQFGPNDLLR**	Detected	Not detected
**GVVGDITDEELLR**	Detected	Detected
**INQFEPVLAR**	Detected	Detected
**IPSVWAGQSLSQLDLR**	Detected	Detected
**IPSVWAGQSLSQLDLRGK**	Detected	Not detected
**MHHHHHHSDR**	Detected	Detected
**NVSIVEMK**	Detected	Detected
**NVSIVEMKIPSVWAGQSLSQLDLR**	Detected	Not detected
**QDMNIIAIDDHAER**	Detected	Detected
**QDMNIIAIDDHAERINQFEPVLAR**	Detected	Not Detected
**SLGVPRVIAK**	Detected	Detected
**SLGVPRVIAKVK**	Detected	Not detected
**SQTAKKVLEK**	Not detected	Detected
**TAGIDTCDTVVVATGENLESSVLAVMHCK**	Detected	Not detected
**TIGILGLGIFGSSVLTALAK**	Detected	Not Detected
**VIAKVKSQTAK**	Detected	Detected
**YNLNVLGFR**	Detected	Detected

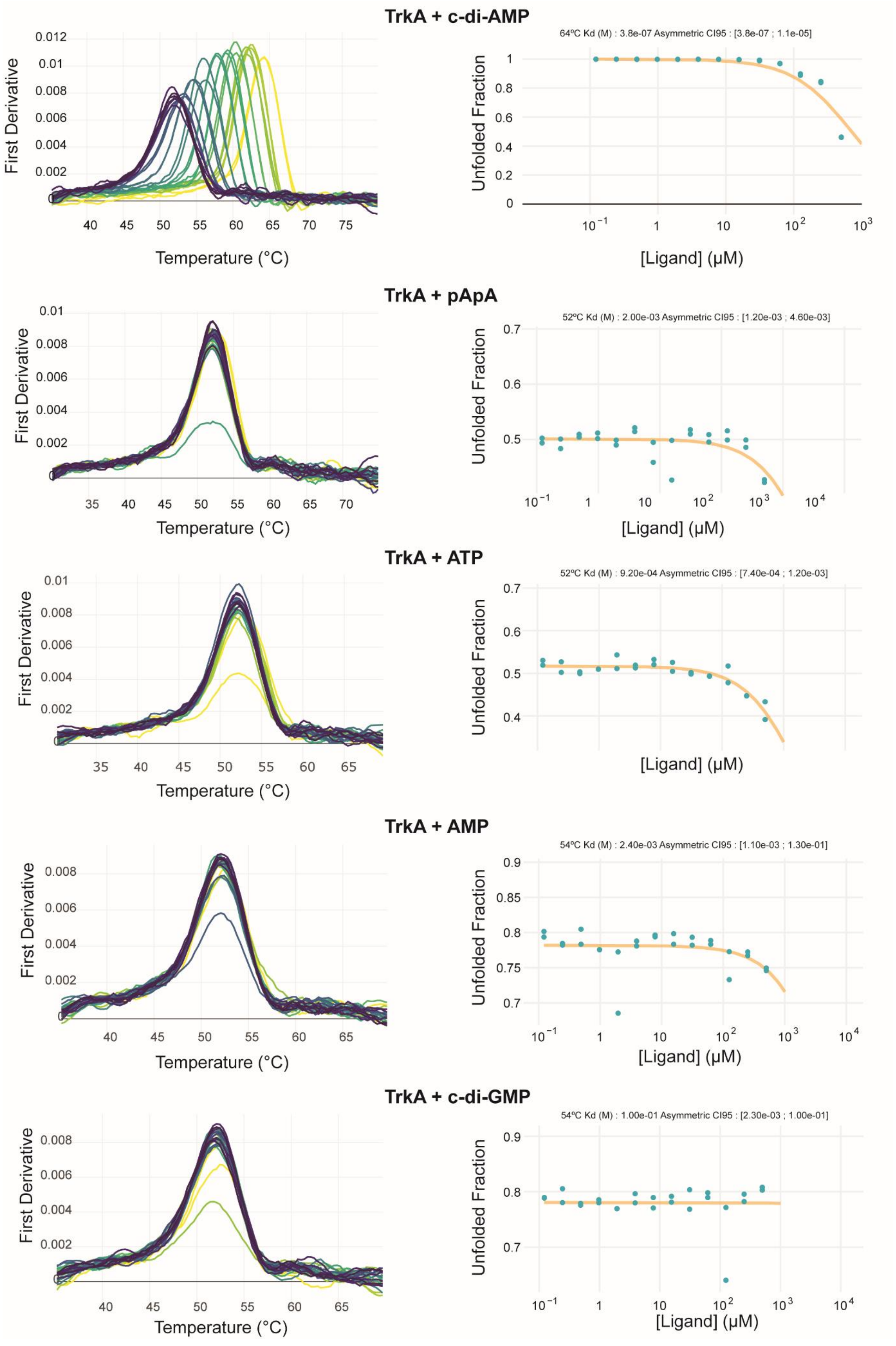
TrkA + c-di-AMP
64ºC Kd (M) : 3.8e-07 Asymmetric CI95 : [3.8e-07 ; 1.1e-05]
TrkA + pApA
52ºC Kd (M) : 2.00e-03 Asymmetric CI95 : [1.20e-03 ; 4.60e-03]
TrkA + ATP
52ºC Kd (M) : 9.20e-04 Asymmetric CI95 : [7.40e-04 ; 1.20e-03]
TrkA + AMP
54ºC Kd (M) : 2.40e-03 Asymmetric CI95 : [1.10e-03 ; 1.30e-01]
TrkA + c-di-GMP
54ºC Kd (M) : 1.00e-01 Asymmetric CI95 : [2.30e-03 ; 1.00e-01]
First Derivative
Temperature (°C)
Unfolded Fraction
[Ligand] (μM)

Figure S4. TrkA binding and affinity for different nucleotides. NanoDSF analysis of purified recombinant TrkA binding to c-di-AMP, pApA, ATP, AMP or c-di-GMP. The graphs in the column to the left show TrkA melting curves with different ligands. In each experiment a negative control with TrkA protein alone was included (Tm_{TrkA} = 51.85°C). The graphs in the column to the right show the isothermal fitting curves of nanoDSF data for $\Delta C_p = 0$ at chosen temperatures estimated using FoldAffinity (Niebling et al. 2021, Burastero et al. 2021). The experiment was performed in duplicate for each protein-ligand combination. Shown graphs are representative results from one experiment.

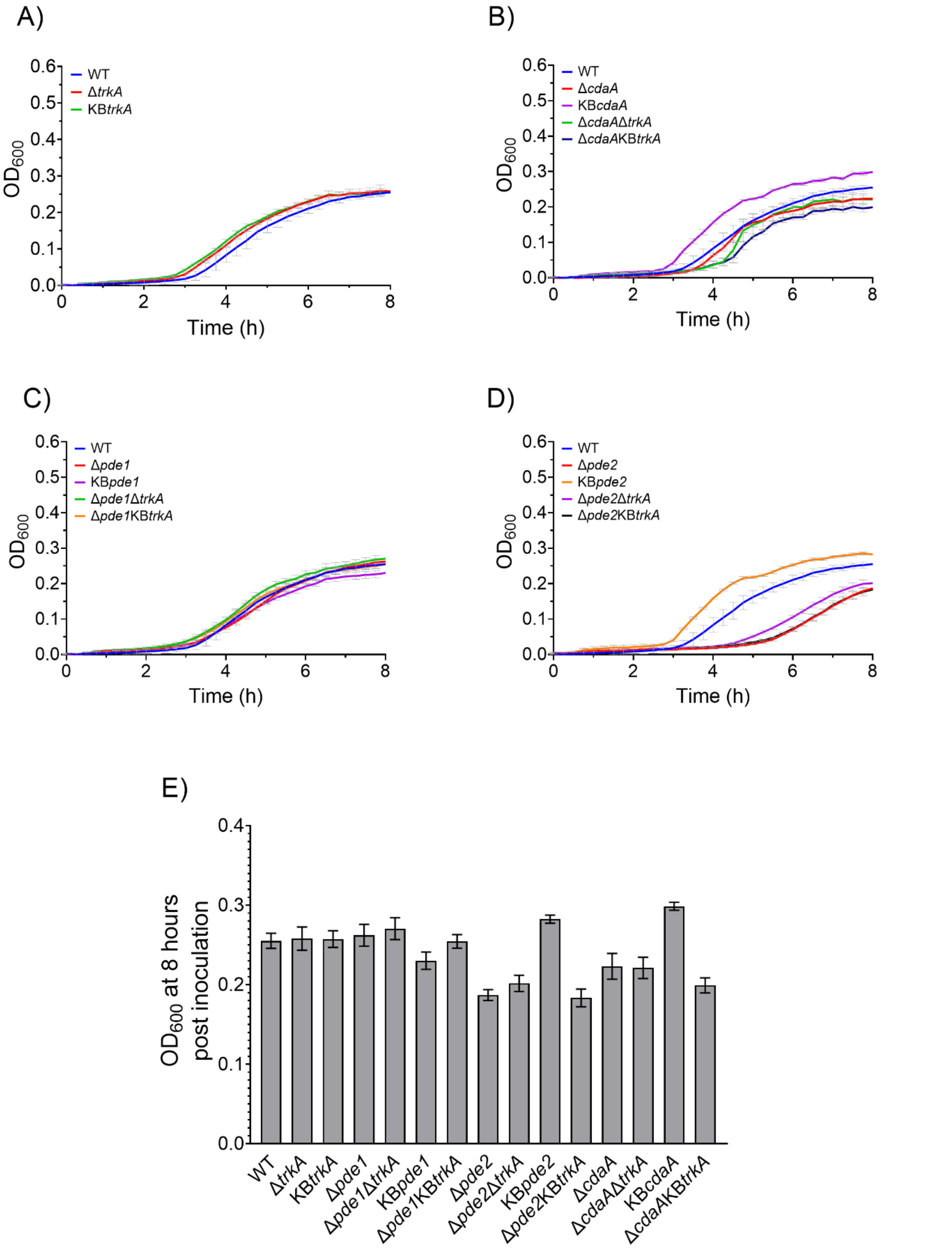

Figure S5. Deletion of trkA does not affect S. mitis growth in TSB. (A) - (D) The growth curves show the mean OD-values collected from two independent experiments with three technical replicates in each. **(E)** The bar graph with OD-values at 8 hours post-inoculation. In all figures, the error bars represent standard deviation.

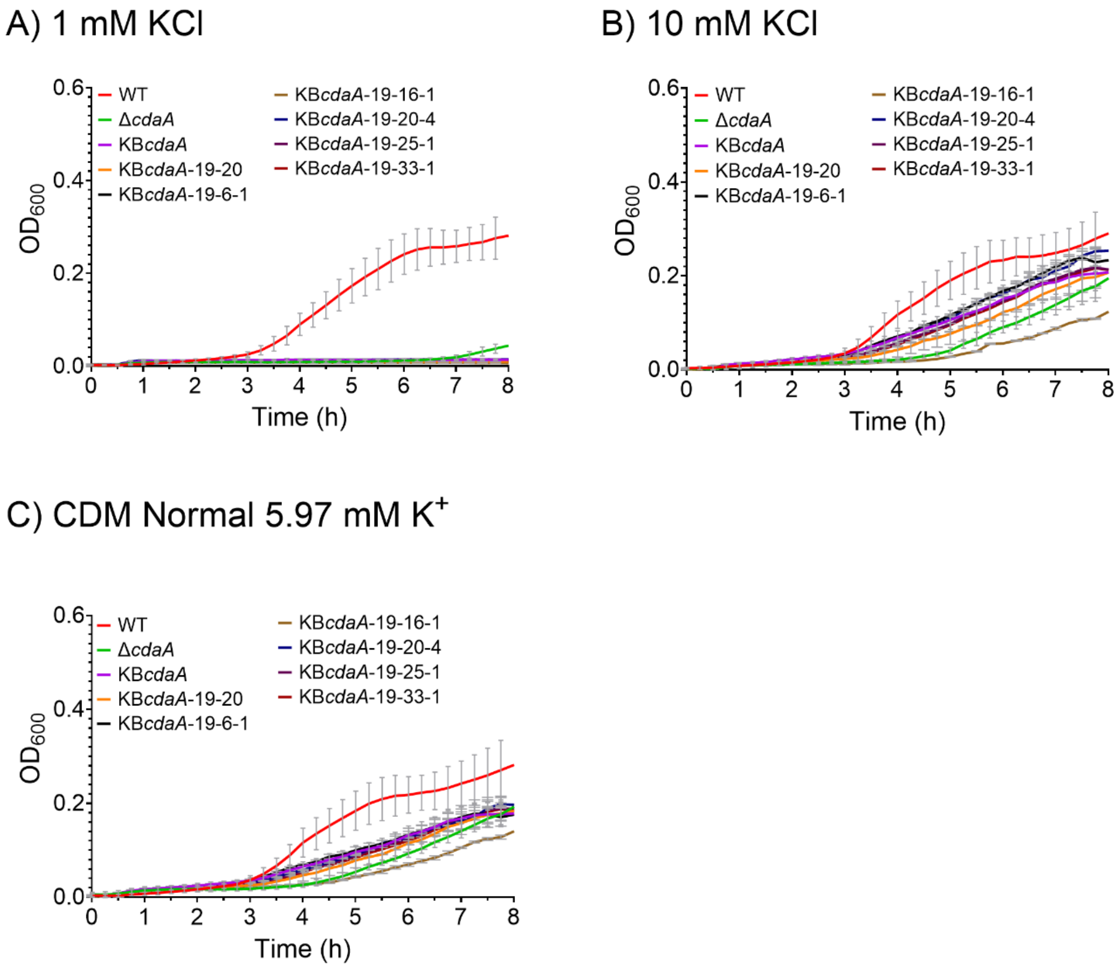

Figure S6. Growth of *S. mitis* Δ*cdaA* and multiple clones of KB*cdaA* in CDM medium with different concentrations and sources of potassium. The growth curves were plotted using representative data from two independent experiments. All the *S. mitis* strains were grown in CDM medium with either **(A)** 1 mM, **(B)** 10 mM KCl, or **(C)** in normal CDM medium with 5.97 mM K^+.

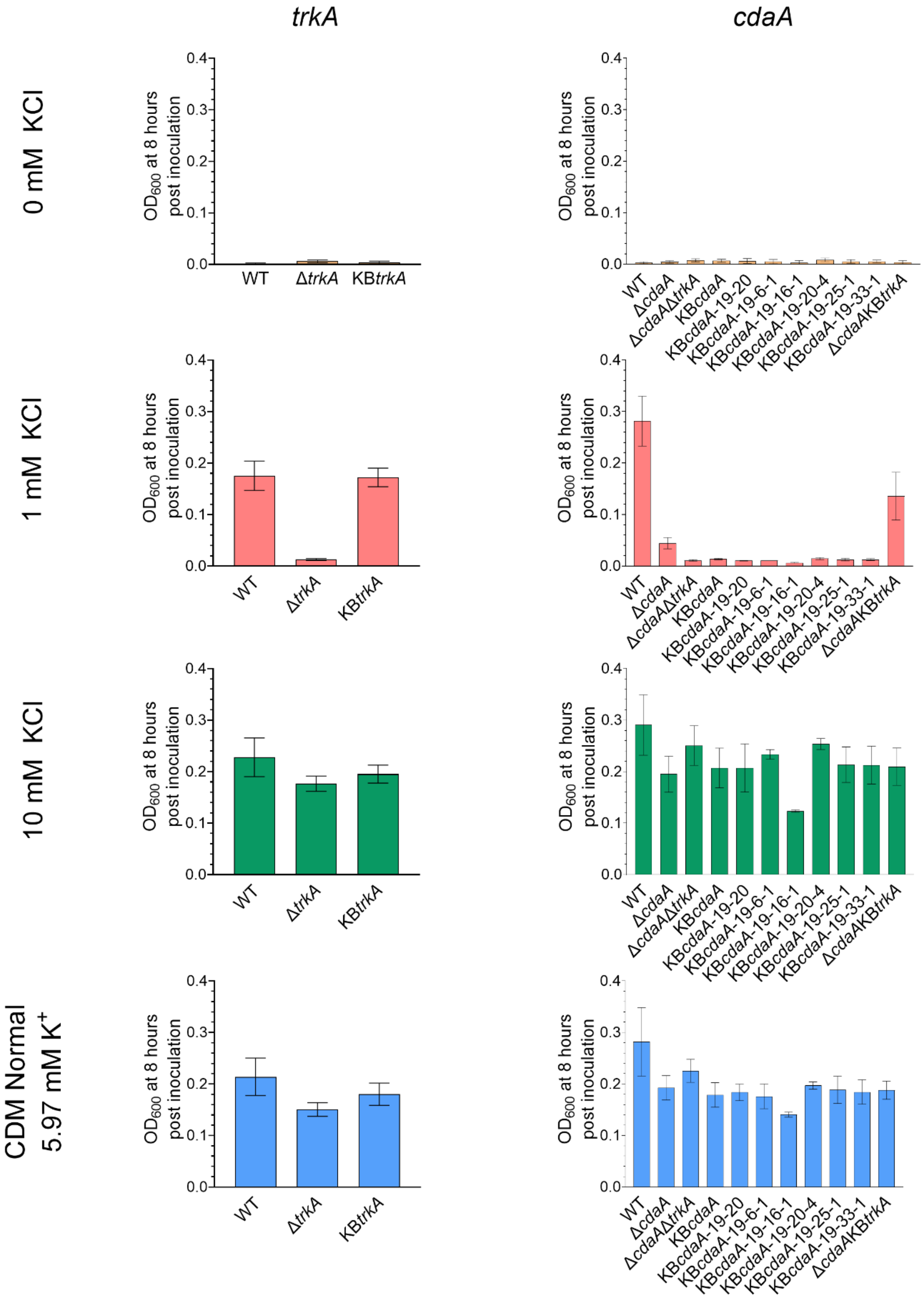

Figure S7a. Growth of *S. mitis* strains in CDM medium with different concentrations and sources of potassium at 8 hours post inoculation. Bar graphs represent data collected at 8 hours post inoculation. Graphs show the complete data collected from 2 independent experiments with 2 technical replicates each. The error bars represent the standard deviation.

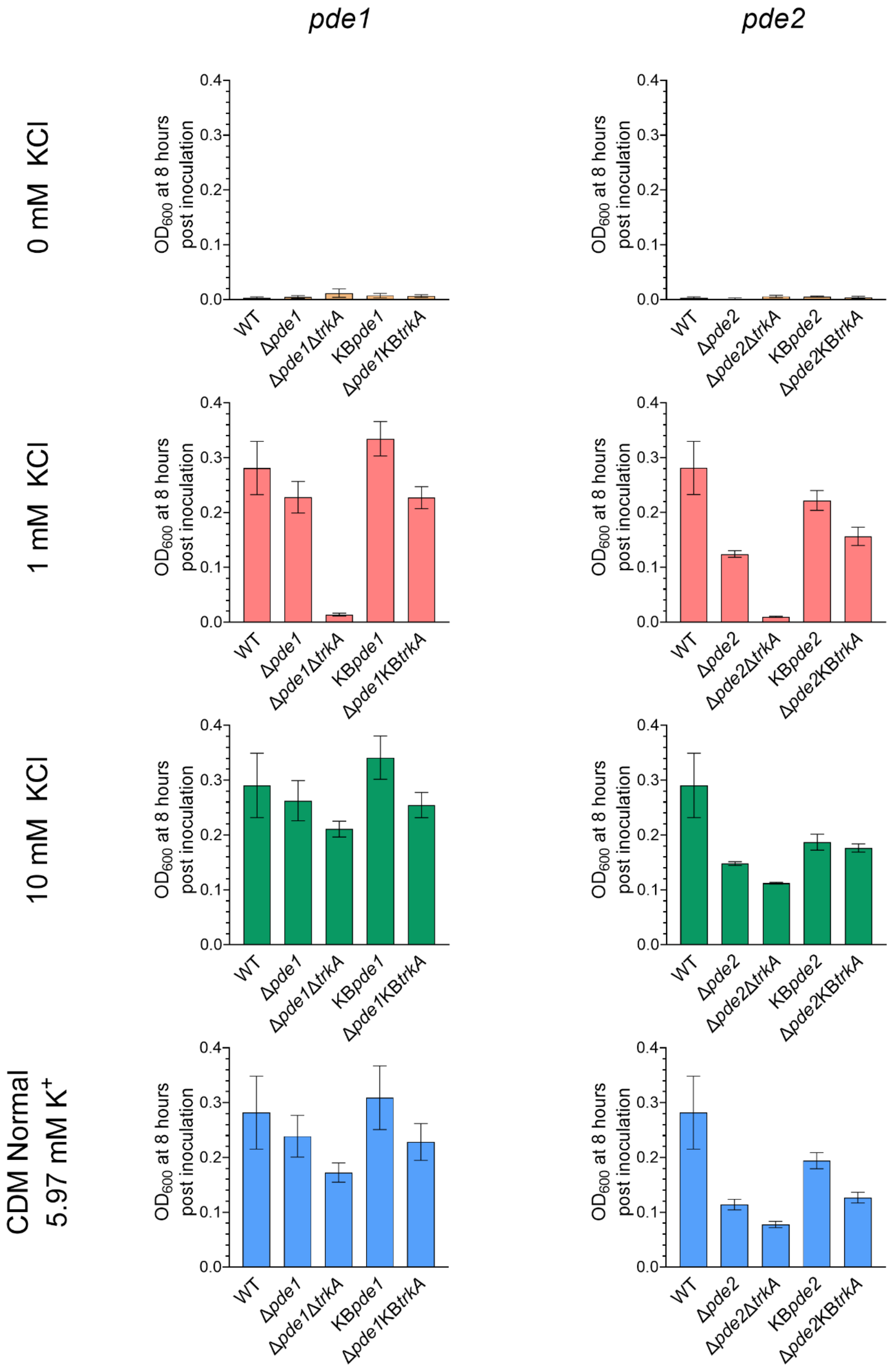

Figure S7b. Growth of *S. mitis* strains in CDM medium with different concentrations and sources of potassium at 8 hours post inoculation. Bar graphs represent data collected at 8 hours post inoculation. Graphs show the complete data collected from 2 independent experiments with 2 technical replicates each. The error bars represent the standard deviation.

Table S6. Whole Genome Sequencing results of the constructed *S. mitis* Δ*cdaA* and KB*cdaA* strains.

Legend: ● Missense variant; ○ Stop gained; ◙ Frameshift variant; ® Synonymous variant

Mutated gene name	Gene product	Effect	Δ*cdaA*	KB *cdaA*	KB *cdaA*-19-20	KB *cdaA*-19-16-1	KB *cdaA*-19-20-4	KB *cdaA*-19-25	KB *cdaA*-19-33-1	KB *cdaA*-19-6-1
**NA**	potassium transporter Trk	missense_variant c.866G>C p.Gly289Ala; frameshift_variant c.608delT p.Val203fs; stop_gained c.268C>T p.Gln90*		●	◙	○	○	◙	○	◙
**ireB**	IreB family regulatory phosphoprotein	missense_variant c.111C>A p.Asn37Lys					●			
**spx**	transcriptional regulator Spx	missense_variant c.214G>A p.Glu72Lys	●	●	●	●	●	●	●	●
**wbbJ**	sugar O-acetyltransferase	synonymous_variant c.177C>A p.Ile59Ile					®			
**NA**	hypothetical protein	missense_variant c.427G>A p.Val143Ile		●						
**mptD**	MptD family putative ECF transporter S component	missense_variant c.425G>A p.Gly142Glu	●	●	●	●	●	●	●	●
**deoC**	Deoxyribose-phosphate aldolase	missense_variant c.46G>C p.Ala16Pro	●	●	●	●	●	●	●	●
**tenA**	TenA family protein	missense_variant c.80C>G p.Ala27Gly			●					
**sufC**	Fe-S cluster assembly ATPase SufC	missense_variant c.499G>T p.Ala167Ser			●	●	●	●	●	●
**cdaR**	CdaR family protein	missense_variant c.290T>C p.Leu97Ser			●					
**rpiR**	MurR/RpiR family transcriptional regulator	missense_variant c.493A>C p.Thr165Pro				●				
**nupQ**	ABC transporter permease	synonymous_variant c.240T>A p.Gly80Gly						®		
**NA**	NADH:quinone reductase	missense_variant c.379G>A p.Glu127Lys	●	●	●	●	●	●	●	●

(Continued on next page)

Mutated gene name	Gene product	Effect	Δ*cdaA*	KB *cdaA*-19-9	KB *cdaA*-19-20	KB *cdaA*-19-16-1	KB *cdaA*-19-20-4	KB *cdaA*-19-25	KB *cdaA*-19-33-1	KB *cdaA*-19-6-1
**pfkA**	ATP-dependent 6-phosphofructokinase	missense_variant c.637A>C p.Lys213Gln					●			
**trpB**	tryptophan synthase subunit beta	synonymous_variant c.291C>A p.Ala97Ala	®	®	®	®	®	®	®	®
**pepS**	Aminopeptidase PepS	missense_variant c.273C>A p.Asn91Lys	●	●	●	●	●	●	●	●
**NA**	glutamyl-tRNA synthetase	missense_variant c.623C>T p.Ala208Val			●			●		●
**tlyC**	hemolysin family protein	missense_variant c.302C>G p.Thr101Arg	●	●	●	●	●	●	●	●
**NA**	Y-family DNA polymerase	stop_gained c.880A>T p.Lys294*			○			○		○
**NA**	hypothetical protein	missense_variant c.1270G>T p.Asp424Tyr					●			
**sfcA**	NAD-dependent malic enzyme	missense_variant c.1570G>A p.Glu524Lys				●	●		●	
**celB**	lactose-specific PTS transporter subunit EIIC	synonymous_variant c.349T>C p.Leu117Leu				®	®		®	
**mdlB**	ABC transporter ATP-binding protein/permease	missense_variant c.1135A>G p.Ile379Val					●			
**uup**	ABC-F family ATP-binding cassette domain-containing protein	synonymous_variant c.1203G>A p.Leu401Leu								®
**smc**	chromosome segregation protein SMC	missense_variant c.2692G>A p.Asp898Asn	●	●	●	●	●	●	●	●
**NA**	hypothetical protein	missense_variant c.4647_4649delAAAinsTAC p.Asn1550Thr		●						

(Continued on next page)

Mutated gene name	Gene product	Effect	Δ*cdaA*	KB *cdaA*-19-9	KB *cdaA*-19-20	KB *cdaA*-19-16-1	KB *cdaA*-19-20-4	KB *cdaA*-19-25	KB *cdaA*-19-33-1	KB *cdaA*-19-6-1
**NA**	hypothetical protein	synonymous_variant c.5199_5208delCAACCCACTTinsTAATCCGTTG p.1737		®						
**NA**	hypothetical protein	synonymous_variant c.5217T>A p.Val1739Val		®						

Table S7. *S. mitis* isolates used for Whole Genome Sequencing and their accession numbers.

Strain	Isolate	Accession number (ENA)
**Streptococcus mitis**	CCUG31611 (Wild Type)	ERS23831685
**Streptococcus mitis**	Δ*cdaA*	ERS23831686
**Streptococcus mitis**	KB*cdaA*-19-9	ERS23831687
**Streptococcus mitis**	KB*cdaA*-19-20	ERS23831688
**Streptococcus mitis**	KB*cdaA*-19-16-1	ERS23831690
**Streptococcus mitis**	KB*cdaA*-19-20-4	ERS23831691
**Streptococcus mitis**	KB*cdaA*-19-25	ERS23831692
**Streptococcus mitis**	KB*cdaA*-19-33-1	ERS23831693
**Streptococcus mitis**	KB*cdaA*-19-6-1	ERS23831689

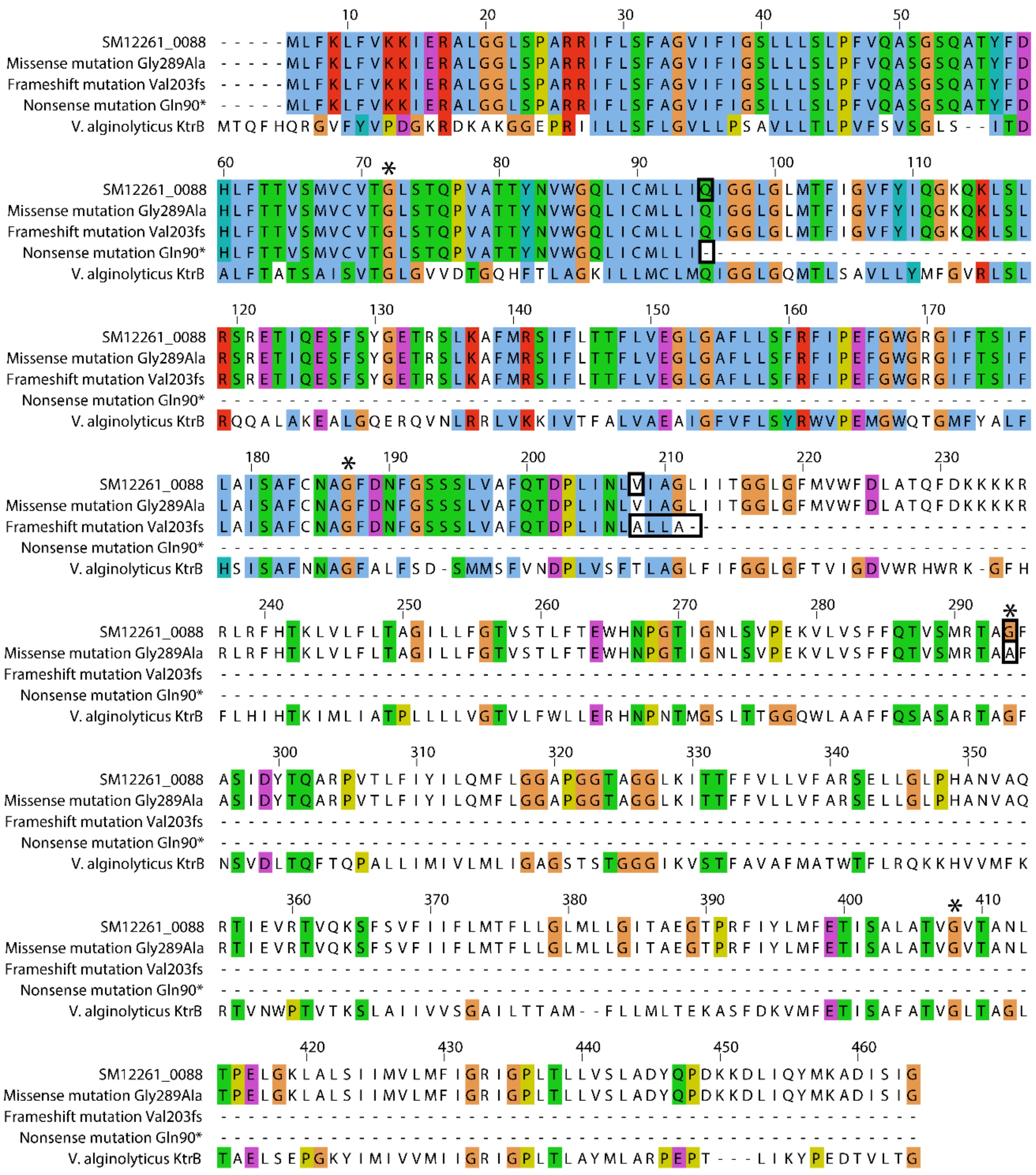

Figure S8. Sequence alignment of potassium transporter proteins. Aligned amino acid sequences of the putative potassium transporter from *S. mitis* CCUG31611 (SM12261_0088) and *V. alginolyticus* KtrB. Conserved amino acid residues are colored and the four conserved glycine residues forming the selectivity filter in *V. alginolyticus* KtrB are indicated with asterisks above the alignment. The original amino acids and their mutated versions in the SM12261_0088 sequence are marked with black boxes.

References:

Burastero, O., S. Niebling, L. A. Defelipe, C. Gunther, A. Struve & M. M. Garcia Alai (2021) eSPC: an online data-analysis platform for molecular biophysics. *Acta Crystallogr D Struct Biol,* 77**,** 1241-1250.

Niebling, S., O. Burastero, J. Burgi, C. Gunther, L. A. Defelipe, S. Sander, E. Gattkowski, R. Anjanappa, M. Wilmanns, S. Springer, H. Tidow & M. Garcia-Alai (2021) FoldAffinity: binding affinities from nDSF experiments. *Sci Rep,* 11**,** 9572.
